# The spatial dissimilarities and connections of the microbiota in the upper and lower respiratory tract of beef cattle

**DOI:** 10.3389/fcimb.2023.1269726

**Published:** 2023-11-06

**Authors:** Zhihao Zhang, Chengqian Zhang, Yikai Zhong, Shuli Yang, Feilong Deng, Ying Li, Jianmin Chai

**Affiliations:** ^1^ Guangdong Provincial Key Laboratory of Animal Molecular Design and Precise Breeding, College of Life Science and Engineering, Foshan University, Foshan, China; ^2^ Division of Agriculture, Department of Animal Science, University of Arkansas, Fayetteville, AR, United States

**Keywords:** respiratory microbiota, bovine respiratory disease, geography, oral cavity, nostrils, nasopharynx, lung

## Abstract

Bovine respiratory disease (BRD) causes morbidity and mortality in cattle. The critical roles of the respiratory microbiota in BRD have been widely studied. The nasopharynx was the most popular sampling niche for BRD pathogen studies. The oral cavity and other niches within the respiratory tract, such as nostrils and lung, are less assessed. In this study, oropharyngeal swabs (OS), nasal swabs (NS), nasopharyngeal swabs (NP), and bronchoalveolar lavage (BAL) were collected from calves located in four countries and analyzed for investigation of the dissimilarities and connections of the respiratory microbiota. The results showed that the microbial diversity, structure, and composition in the upper and lower respiratory tract in beef cattle from China, the USA, Canada, and Italy were significantly different. The microbial taxa for each sampling niche were specific and associated with their local physiology and geography. The signature microbiota for OS, NS, NP, and BAL were identified using the LEfSe algorithm. Although the spatial dissimilarities among the respiratory niches existed, the microbial connections were observed in beef cattle regardless of geography. Notably, the nostril and nasopharynx had more similar microbiomes compared to lung communities. The major bacterial immigration patterns in the bovine respiratory tract were estimated and some of them were associated with geography. In addition, the contribution of oral microbiota to the nasal and lung ecosystems was confirmed. Lastly, microbial interactions were characterized to reveal the correlation between the commercial microbiota and BRD-associated pathogens. In conclusion, shared airway microbiota among niches and geography provides the possibility to investigate the common knowledge for bovine respiratory health and diseases. In spite of the dissimilarities of the respiratory microbiota in cattle, the spatial connections among these sampling niches not only allow us to deeply understand the airway ecosystem but also benefit the research and development of probiotics for BRD.

## Introduction

Bovine respiratory disease (BRD), causing huge economic costs worldwide, is one of the most common diseases in beef cattle (Chai et al., 2022a). The respiratory microbiota in cattle associated with disease has been confirmed, and several bacterial pathogens in BRD have been identified, such as *Mycoplasma bovis*, *Mannheimia haemolytica*, *Histophilus somni*, and *Pasteurella multocida* (Nicola et al., 2017; McMullen et al., 2020b). However, since the physiological and biochemical environments of different niches along the bovine respiratory tract result in the dissimilarities of microbial compositions, these opportunistic pathogens do not have great agreement with BRD onset (Dickson et al., 2016; Cirone et al., 2019). Moreover, the variation of geographic climate causes changes in the respiratory microbiota even in healthy cattle (Chai et al., 2022b), resulting in challenges to understanding the microbial ecology, and identifying pathogens and probiotics. Therefore, a deep investigation into the spatial dissimilarity and connection of the respiratory microbiota in healthy cattle is necessary and provides insights into bovine respiratory disease.

Nasopharynx is the most frequent sampling niche used to investigate bovine respiratory microbiota (Holman et al., 2015; Amat et al., 2019; Holman et al., 2019; McMullen et al., 2020a; Zeineldin et al., 2020). Notably, the upper airway microbiota associated with respiratory diseases has also been widely investigated in humans (Wang et al., 2020; Losol et al., 2021; Wang et al., 2022). Microbiota colonizing in other niches, such as nostrils and lungs, are less studied but also important as the physiological environment of the whole respiratory tract is changed in BRD cattle (Man et al., 2017; Fahkrajang et al., 2021). A study found different microbial structures and dominant bacteria between the upper and lower respiratory tract in Piedmontese calves (Nicola et al., 2017), indicating that niche physiology influences the microbial community. Thus, investigation of nasal and lung microbiota in healthy or BRD calves is also necessary to elucidate respiratory homeostasis or dysbiosis. It was previously suggested that bovine nasal bacterial communities provide a potential pen-side diagnostic testing for BRD (Centeno-Martinez et al., 2022). In the meantime, the dispersal of the microbiota within the respiratory system exists as shared bacterial taxon among different niches of the bovine respiratory tract was observed (McMullen et al., 2020a), and oral microbiota being one of the main sources of lung community was reported in humans (Venkataraman et al., 2015) and determines two microbiota pneumo-types associated with health status (Zhang et al., 2022). In cattle, the pathogens in the lungs could be from the nostrils or mouth based on the theory that “disease enters by the mouth”. Similarly, the genera associated with common BRD pathogens such as *Mycoplasma*, *Mannheimia*, and *Pasteurella* are observed in the nostrils and oral cavity in healthy and BRD cattle (Nicola et al., 2017; McMullen et al., 2020a). All these imply that the microbial composition of the nostril and mouth is critical to the lung microbiome community in cattle. However, to our knowledge, there are fewer studies to specifically investigate spatial dissimilarity and connection of the bovine respiratory microbiota.

In this study, bovine respiratory samples from China, the United States, Canada, and Italy were collected to estimate the geographic effects, and the dissimilarities among niches, including oropharynx, nostrils, nasopharynx, and lungs were determined. Notably, the microbial connection or migration from the upper airway or mouth to the lungs in bovines were first confirmed and characterized, which provides the fundamental knowledge for understanding the bovine respiratory system.

## Materials and methods

The experiment protocol was approved by the Animal Ethics and Humane Animal Care of the Foshan University.

### Sample collection

A total of thirteen steers, twelve to eighteen months old, of two breeds (Gayal (Bos gaurus frontalis): n=5 and Zebu (Bos taurus indicus): n=8) from Yunnan province, China were selected in October 2022 (**Supplementary Table S1**). All steers were clinically healthy and did not receive any recorded therapeutic or prophylactic antibiotic treatments. In this study, all calves were sampled using oropharyngeal swabs (OS), nasal swabs (NS), nasopharyngeal swabs (NP), and bronchoalveolar lavage (BAL). OS was collected by swirling two Puritan Opti-Swabs (Puritan Medical Products Co. LLC, Guilford, Maine) in the end and over the tongue until saturation. NS were collected by swirling swabs in the right nostril until saturation. NP was collected by inserting a double guarded culture swab (Jorgensen Labs, Loveland, Colorado) up the nares until reaching the nasopharynx where the swab was advanced through the guard, rotated against the nasopharyngeal mucosa, then retracted back into the guard and removed from the nares. For a BAL sample, a tube (MILA International, Florence, KY) was passed through the nares, guided through the larynx into the trachea, and advanced until resistance was met. Sterile 0.9% saline was administered in aliquots of 60 ml (up to 240 ml) and aspirated. All samples were transported on dry ice to the laboratory, and stored at −80°C pending further processing.

Simultaneously, our study collected public datasets published by Nicola et al. (Nicola et al., 2017), Holman et al. (Holman et al., 2017; Holman et al., 2018; Holman et al., 2019), McMullen et al. (McMullen et al., 2020a), Zeineldin et al. (Zeineldin et al., 2017b) and Centeno−Martinez et al. (Centeno-Martinez et al., 2022) (**Supplementary Table S2**). These datasets contained 400 respiratory tract samples from healthy calves, which contained 18 OS samples, 160 NS samples, 87 NP samples, and 135 BAL samples, collected in different countries. In the meantime, these calves were from different elevations which was described in **Supplementary Table S1**. All sequences were downloaded from the NCBI SRA database.

### DNA extraction and next-generation sequencing

All samples were thawed on ice and DNA was extracted using a commercial DNA Kit (Omega Bio-tek, Norcross, GA, U.S.) according to the manufacturer’s instructions. Sterile Opti-Swab Amies buffer was taken through the extraction process representing a negative control. Total DNA quality was analyzed using a NanoDrop 2000 UV spectrophotometer (ThermoFisher, Waltham, MA, USA) and 1% agarose gel electrophoresis. The V3 - V4 region of the bacterial 16S ribosomal RNA genes were amplified by PCR (95°C for 3 min, followed by 30 cycles at 98°C for 20 s, 58°C for 15 s, and 72°C for 20 s and a final extension at 72°C for 5 min) using indexes and adaptor-linked universal primers (338F: ACTCCTACGGGAGGCAGCA; 806R: GGACTACHVGGGTWTCTAAT). PCR reactions were performed in 30 μL mixtures containing 15 μL of 2 × KAPA Library Amplification Ready Mix, 1 μL of each primer (10 μM), and 50 ng of template DNA and ddH2O. All PCR products were normalized and quantified by a Qubit 2.0 Fluorometer (Thermo Fisher Scientific, Waltham, MA, USA). Amplicon libraries were mixed using all qualified products and sequenced with an Illumina HiSeq platform at Biomarker Technologies Corporation (Beijing, China).

### Sequence processing

The software package QIIME2 (version 2020.6) (Bolyen et al., 2019) was applied to analyze the next-generation sequencing data from the Illumina MiSeq platform. After fastq files were imported together into QIIME2, the Deblur program was used to process the raw reads. Deblur, a novel sub-operational-taxonomic-unit approach, uses error profiles to obtain putative error-free sequences, resulting in high-quality amplicon sequence variants (ASVs) (Amir et al., 2017). After the quality filtering step was completed, high-quality reads were normalized to minimize the effects of sequencing depth on alpha and beta diversity measures. The Bray-Curtis and Jaccard distance metrics were calculated to investigate the dissimilarities in community structure. The ANalysis Of SIMilarity was employed to compare the significance of beta diversity. Then, clean reads were classified using the Greengenes reference database (13-8 version) (DeSantis et al., 2006) which classifies 99% similarity. A bacterial ASVs table was generated using the QIIME2 command.

### Statistical analyses

Determination of alpha and beta diversity was performed in the QIIME2 platform. Alpha diversity (Shannon index) was calculated using the Kruskal-Wallis test to explore the difference between different groups. Beta diversity was evaluated using Bray-Curtis (Bray and Curtis, 1957) and Jaccard (Chao et al., 2005) distances. The analysis of similarities (ANOSIM) was performed to calculate the P value and correlation coefficient (R-value), and explored similarity and dissimilarity between members of different groups. For all analyses, statistical significance was determined at p < 0.05. The algorithm of linear discriminant analysis (LDA) effect size (LEfSe) was performed using the non-parametric Kruskal–Wallis and pair Wilcoxon rank sum tests to determine the features with significantly different abundances between groups. The LEfSe’s threshold on the logarithmic score of LDA was set to 4.0, the remaining settings were default parameters. All figures were generated with the ggplot2 and pheatmap packages in R (Wickham, 2011).

To analyze the microbial associations among niches (oropharynx, nostrils, nasopharynx, and lung), we first detected the shared bacterial taxa among them. Then, using Pearson correlation between niches, the microbial community similarity was calculated by accounting for both the rank order of ASVs and the magnitude of relative abundances. Subsequently, the important taxa were deeply analyzed to reveal micro aspiration.

## Results

### Sample characteristics and sequencing analysis

A total of 441 samples from our lab and publicly available datasets were included in this study. The characteristics of the samples are summarized in **Table S2**. The beef cattle were from four geographic locations, including China, the USA, Canada, and Italy. The respiratory microbial samples were collected from oropharynx, nostrils, nasopharynx, and lung using oral swabs (OS), nasal swabs (NS), nasopharyngeal swabs (NPS), and bronchoalveolar lavage (BAL). A total of 25,991,588 high-quality reads were generated with an average of 58,937 reads per sample. After rarefaction of sample reads to 2000, a total of 19,295 ASVs from 428 samples were included for downstream analysis, which identified 644 genera. The other thirteen samples with sequence read numbers below 2000 were excluded from further analysis.

### The respiratory microbiota in beef cattle is affected by geographic locations and sampling niches

**Figure 1:**
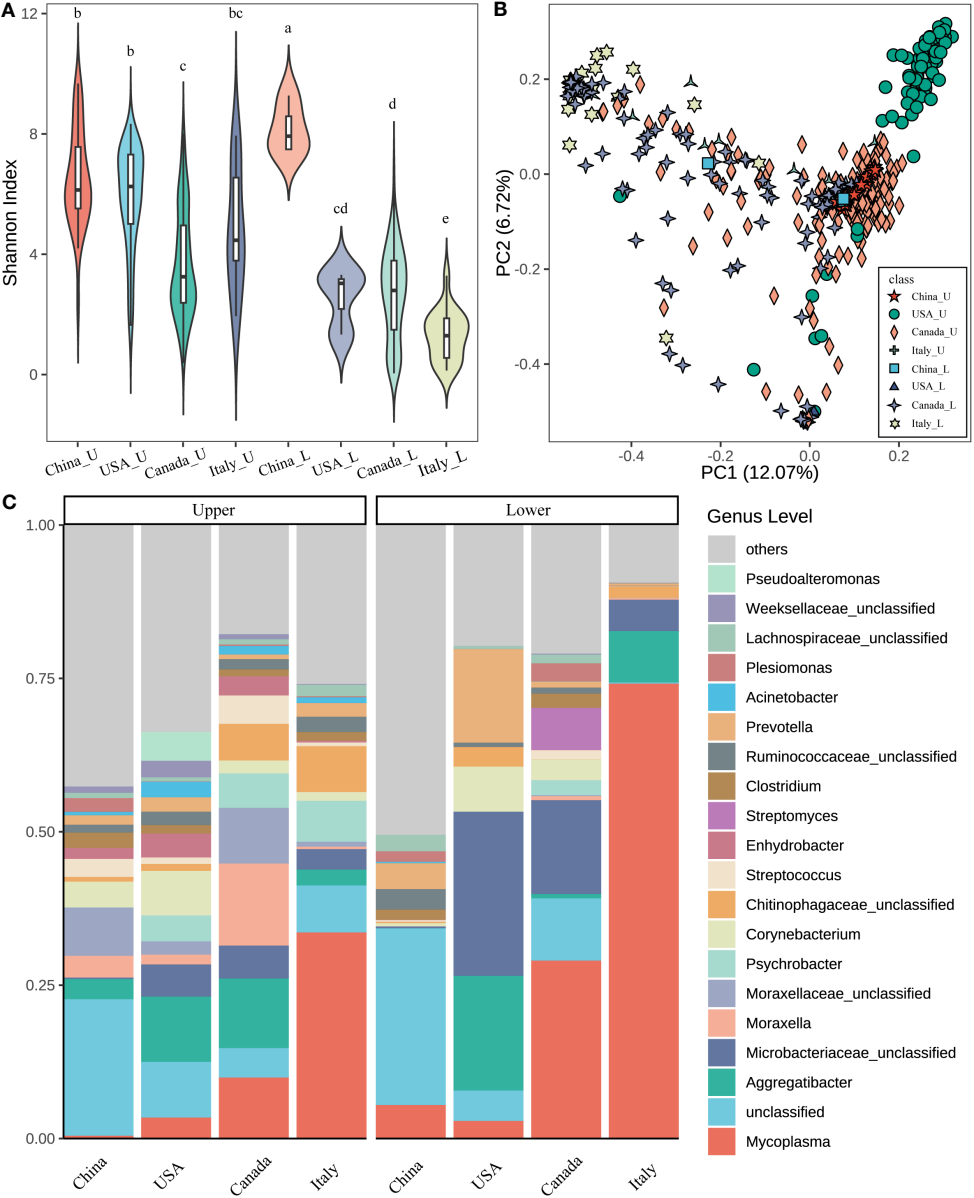
Microbial diversity and structure of the respiratory tract in different countries. **(A)** Alpha diversity (Shannon index) of the upper and lower respiratory tract in different countries. The letters on the box, which was the same between different groups mean that the difference is not significant (p > 0.05). On the contrary, the difference is significant (p < 0.05). **(B)** The principal coordinate analysis (PCoA) is based on the beta diversity (Bray–Curtis) of microbes in each sample (a point represents a sample) of different countries. **(C)** The composition of the top 20 microbes of the genus level in the average relative abundance of the respiratory tract in different countries. ***_U means the upper respiratory tract microbes of *** cattle; ***_L means the lower respiratory tract microbes of *** cattle (*** indicates any one of China, USA, Canada, and Italy).

The microbiota from the upper (U) and lower (L) respiratory tract in the beef cattle associated with geography was found. The beef calves from China, the USA, Canada, and Italy showed significant differences in the U and L microbial diversity (**Figure 1A**). The U airway microbiota diversity from Canada was significantly lower than that from China and the USA. The L microbial diversity in cattle from China was the greatest followed by the USA, Canada, and Italy had the least. In the meantime, except in China, the U alpha diversity was significantly higher (p < 0.05) compared to the L in the calves from the same country.

Regarding the beta diversity based on Bray-Curtis distance, the geography affecting the microbial structure in the U and L was also observed (**Figure 1B**). The U airway microbiota in cattle from the USA showed a distinct cluster compared to other countries (ANOSIM, USA vs China: R = 0.767; USA vs Italy: R = 0.625, *P* = 0.001 for both). Similarly, the L airway microbial structure was also influenced by geographic locations. Moreover, the differences between the U and L airway microbiota in the same country were also observed (**Supplementary Table S3**).

Next, the microbial composition in the U and L respiratory tracts of cattle from different countries was estimated. At the phylum level, the dominant bacteria were Proteobacteria (33.99%), Firmicutes (18.90%), Tenericutes (16.07%), Actinobacteria (15.90%), and Bacteroidetes (10.84%) across all samples. Notably, in the U airway, Proteobacteria was greater in China, the USA, and Canada compared to Italy, while Tenericutes was higher in Italy (**Figure S1**). For the L respiratory tract, Tenericutes was higher in Italy followed by Canada, China, and the USA. Higher Actinobacteria were observed in the USA and Canada. In addition, across the four countries, the L airway had lower abundances of Proteobacteria but higher abundances of Tenericutes compared to the U airway.

At the genus level, the five most predominant genera were *Mycoplasma* (19.87%), *Microbacteriaceae unclassified* (7.71%), *Aggregatibacter* (6.95%), *Moraxellaceae unclassified* (2.50%), and *Moraxella* (2.48%) across all samples (**Figure 1C**). The relative abundances of top taxa among the four countries were significantly different. For example, *Mycoplasma* had the highest abundance in the U and L airways of cattle from Italy followed by Canada, the USA, and China. *Aggregatibacter* had high abundance in the U airway of cattle from the USA and Canada, but abundant in the L airway of cattle from the USA and Italy. *Microbaceriaceae unclassified* were greater in both the U and L airways of cattle from the USA, Canada, and Italy. *Moraxella* was enriched in the U airway of cattle from Canada but lower in other niches of cattle from other countries. Moreover, the different microbial compositions between the U and L airways were also observed. For instance, *Mycoplasma* was higher in the L airway of cattle from all four countries, *Microbaceriaceae unclassified* had a similar pattern especially in cattle from the USA and Canada.

**Figure 2:**
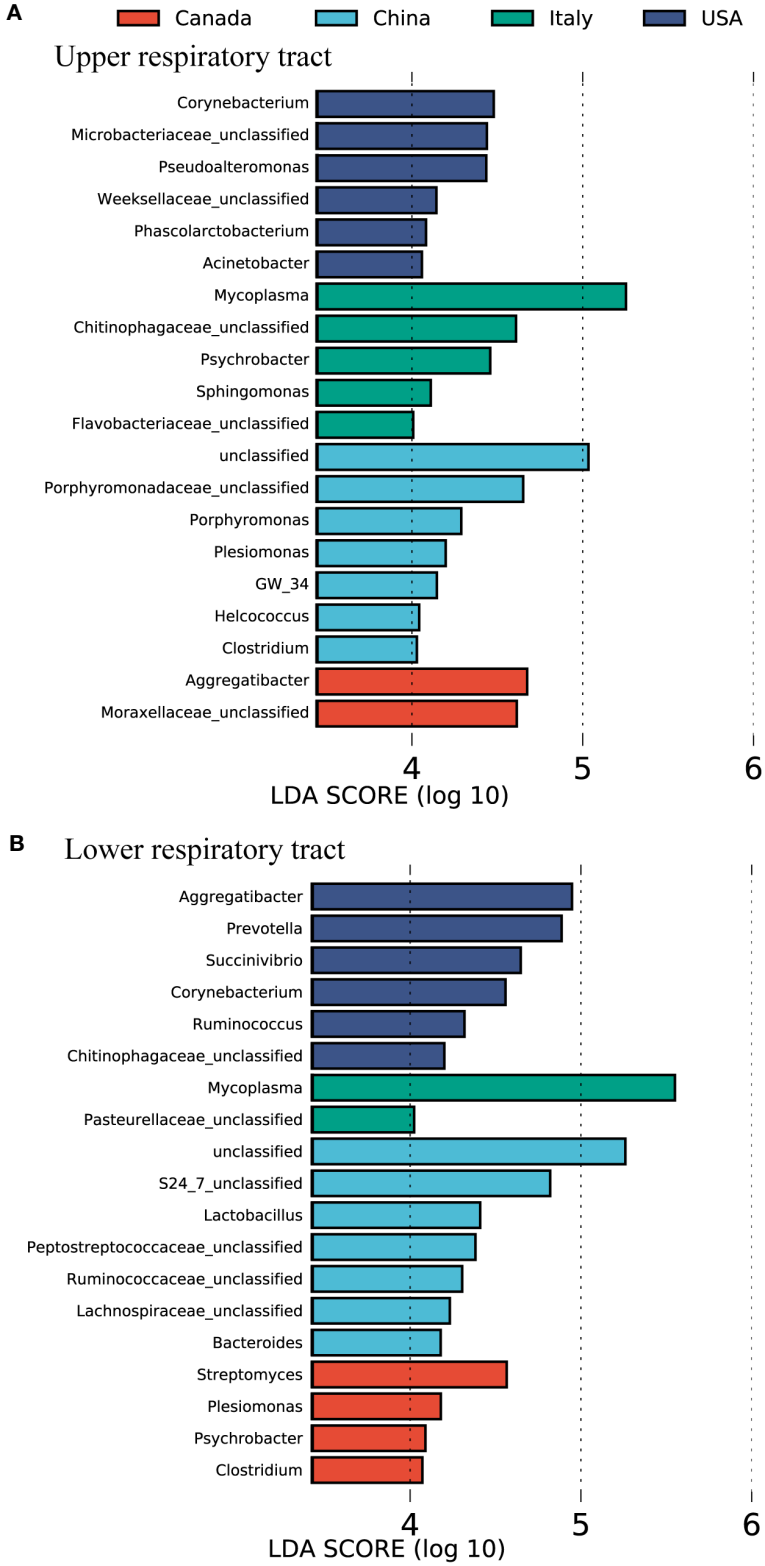
The featured microbes of the calve respiratory tract in different countries identified by LEfSe analysis. **(A)** The featured microbes of the upper respiratory tract identified. **(B)** The featured microbes of the lower respiratory tract identified. Samples were collected from four countries. The general accounting for > 0.1% of the average relative abundance of each genus were selected for LEfSe analysis. Genera in this figure were significant (p < 0.05), had an LDA Score > 4, and set the less strict multi-class analysis, which was considered a significant effect size.

The bovine airway bacterial features influenced by geography were identified by using LEfSe (**Figure 2**). *Porphyromonadaceae unclassified*, *Porphyromonas*, *Plesiomonas*, *Helcococcus*, and *Clostridium* were abundant in the U airway of cattle from China (**Figure 2A**). The U airway of USA cattle had a high abundance of *Corynebacterium*, *Microbacteriaceae unclassified*, *Pseudoalteromonas*, and *Acinetobacter*. The bacteria, including *Aggregatibacter* and *Moraxellaceae unclassified*, were enriched in the U airway of Canadian cattle. *Mycoplasma*, *Chitinophagaceae unclassified*, *Psychrobacter*, and *Sphingomonas* were over-represented in the U airway of cattle from Italy. The same analysis for the L airway microbiota among four countries was also performed (**Figure 2B**). Some gut microbiotas, including *S24_7 unclassified*, *Lactobacillus*, *Peptostreptococcaceae unclassified*, *Ruminococcaceae unclassified*, *Lachnospiraceae unclassified*, and *Bacteroides*, were enriched in the L airway of cattle from China. *Mycoplasma* was over-represented in the lungs of cattle from Italy, and *Aggregatibacter*, *Prevotella*, and *Corynebacterium* were abundant in USA cattle.

### Distribution of the main microbes in different niches

**Figure 3:**
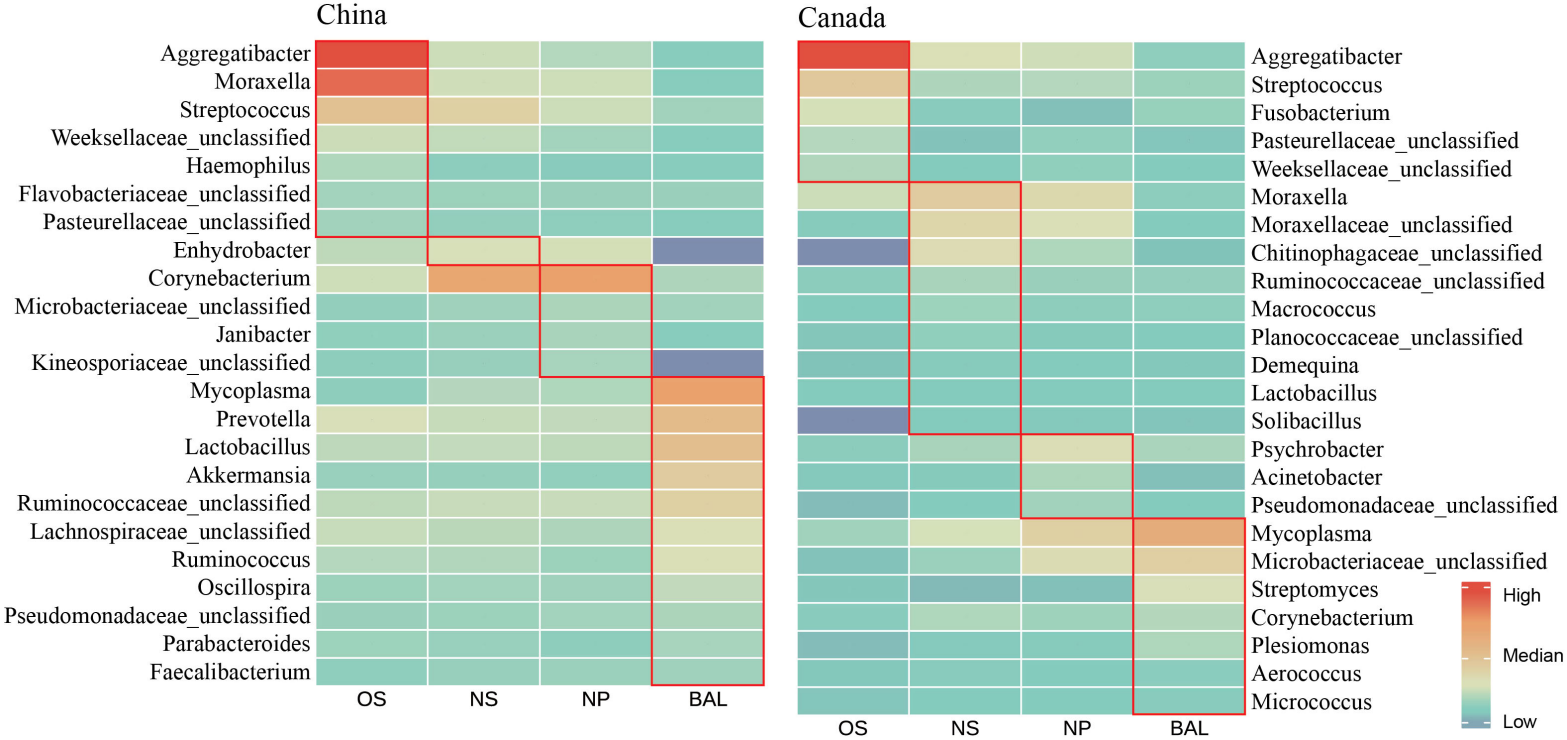
Abundance profile of featured microbes in different ecological niches in the respiratory tract of cattle in China and Canada. The average value of the relative abundance of the single bacteria was compared after making the logarithm in different niches. The red box represents featured microbes that have the highest relative abundance in this niche. The featured microbes were selected based on the results of LEfSe analysis (LDA > 2) across various niches and the significant co-efficient relationship (Pearson rank correlation coefficient > 0.4 or < -0.4) with specific bacteria. OS (Oropharynx); NS (Nasal); NP (Nasopharyngeal); BAL (Bronchoalveolar lavage).

As the physiology of different respiratory niches influences the microbial composition, LEfSe analysis and correlation analysis were performed to screen out the signature microbes for the major niches (OS, NS, NP, and BAL) along the bovine respiratory tract (**Figures 3**, **S2**). In Chinese cattle, *Aggregatibacter*, *Moraxella*, *Streptococcus*, *Weeksellaceae unclassified*, *Haemophilus*, *Flavobacteriaceae unclassified*, and *Pasteurellaceae unclassified* had higher abundances in the OS (**Figure 3**). In the NS, only the *Enhydrobacter* genus was more abundant. In the NP, the abundance of *Corynebacterium*, *Microbacteriaceae unclassified*, *Janibacter*, and *Kineosporiaceae unclassified* was greater. In BAL, bacterial genera, including *Mycoplasma*, *Prevotella*, *Lactobacillus*, *Akkermansia*, *Ruminococcaceae unclassified*, *Lachnospiraceae unclassified*, *Ruminococcus*, *Oscillospira*, *Pseudomonadaceae unclassified*, *Parabacteroides*, and *Faecalibacterium*, were more abundant. In Canadian cattle, *Aggregatibacter*, *Streptococcus*, *Fusobacterium*, *Pasteurellaceae unclassified*, and *Weekselllaceae unclassified* had higher abundance in the OS. In the NS, *Moraxella*, *Moraxellaceae unclassified*, *Chitinophagaceae unclassified*, *Ruminococcaceae unclassified*, *Macrococcus*, *Planococcaceae unclassified*, *Demequina*, *Lactobacillus*, and *Solibacillus* were more abundant. In the NP, the abundance of *Psychrobacter*, *Acinetobacter*, and *Pseudomonadaceae unclassified* was higher. In the BAL, the abundances of *Mycoplasma*, *Microbacteriaceae unclassified*, *Streptomyces*, *Corynebacterium*, *Plesiomonas*, *Aerococcus*, and *Micrococcus* were high.

Moreover, some signature bacteria for one niche were found across countries. For example, *Aggregatibacter* and *Streptococcus* were abundant in the OS of both Chinese and Canadian cattle (**Figure 3**), and they were also identified as OS signatures in the LEfSe outputs using all samples from four countries (**Figure S2**). *Mycoplasma* abundant in the BAL had a similar pattern. Overall, although the niche-specific microbiota was associated with geography, shared microbiotas were found and detected in all four niches.

### The spatial dissimilarities and connections of the microbiota in the respiratory tract of beef cattle

**Figure 4:**
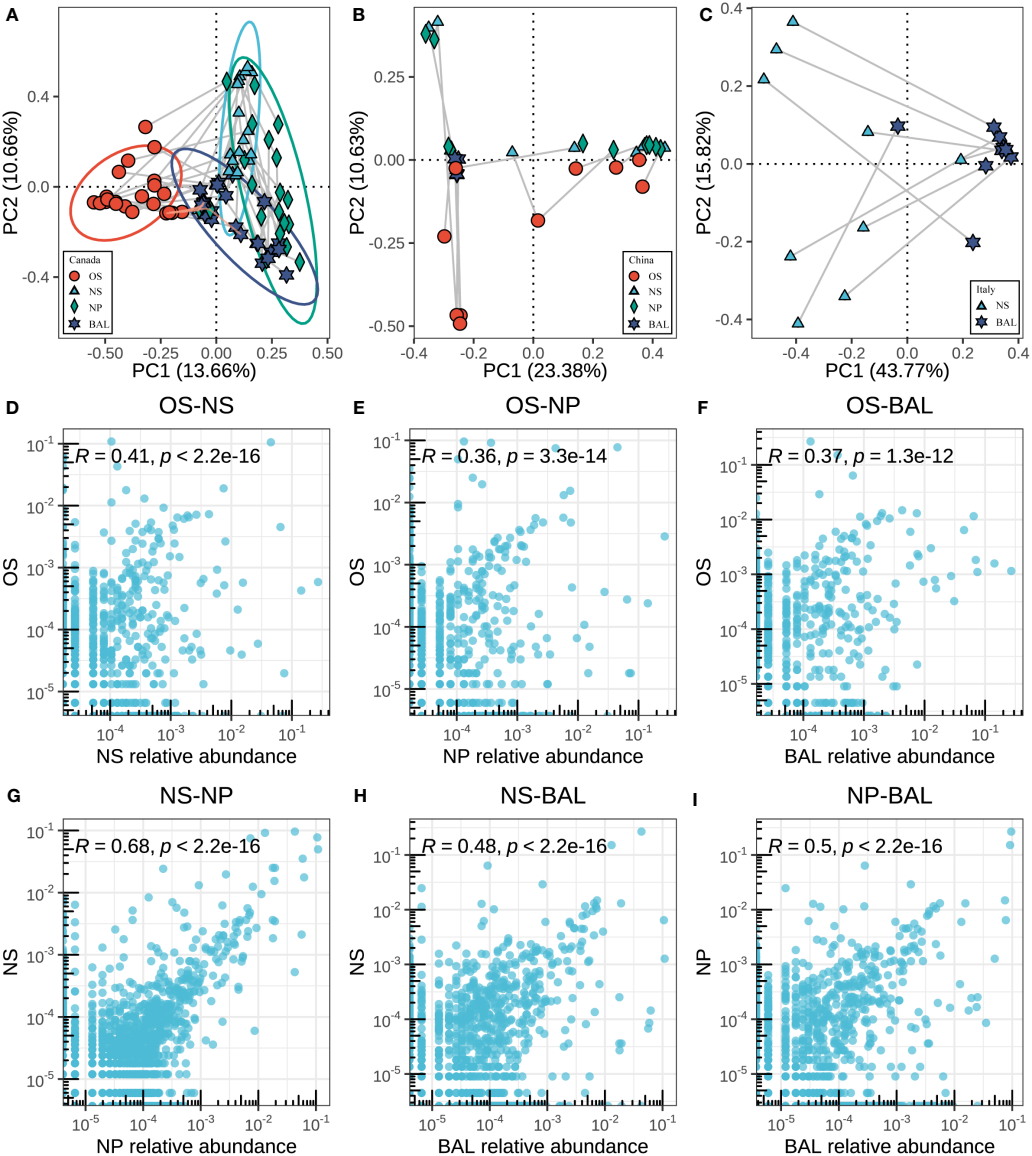
Associations between microbes in different niches of the respiratory tract. **(A–C)** The principal coordinate analysis (PCoA) is based on the beta diversity (Bray–Curtis) of microbes in each sample between different niches in different countries. The Connected points represent samples from the same animal. The length of the line reflects the similarity between samples, and the longer lines represent the lower similarity. **(D–I)** The correlation of the respiratory tract microbes in different niches in Canadian samples. Each point corresponds to the average relative abundance of a feature across all animals for each of the respiratory tract sampling niches. To measure correlation, Pearson’s r was calculated based on the features abundance of two niches.

After the different microbial compositions in the niches along the bovine respiratory tract were characterized, the spatial dynamics of the bovine respiratory microbiota from the same cattle were estimated. When excluding the geographic effects, distinct microbial structures between the oral cavity, upper, and lung were also observed, which had a similar pattern in samples of different countries (**Figures 4A–C**). For example, regarding the beta diversity based on Bray-Curtis distance, in the different geographies, microbiota in different niches showed distinct clusters (**Figures 4A–C**) (ANOSIM, in Canada, NS vs BAL: R = 0.365; in China, NS vs BAL: R = 0.285; in Italy, NS vs BAL: R = 0.529, *P* < 0.05, **Supplementary Table S4**). However, regardless of the countries, bigger differences between NP and BAL were observed compared to NS and BAL (**Supplementary Table S5**). Moreover, oropharyngeal microbiota seemed to be independent of the respiratory microbiota in beef cattle as it showed a large distance from NS, NP, and BAL.

Next, we sought to analyze the microbial associations among niches (oropharynx, nostrils, nasopharynx, and lung) as shared bacteria taxa existed and the anatomical connections may lead to microbial migration within the respiratory systems. We assessed the similarities between niches by measuring the Pearson correlation between sampling niches, thus accounting for both the rank order of ASVs and the magnitude of relative abundances between sampling sites being compared. In the Canadian beef cattle, the correlation between OS vs NS was higher than OS vs NP and OS vs BAL (Pearson, OS vs NS: R = 0.41; OS vs NP: R = 0.36; OS vs BAL: R = 0.37) (**Figures 4D–F**). The NS microbiota was highly correlated with that of the NPS (r=0.68, p<0.001) (**Figure 4G**). The correlation between NPS and BAL microbiota (r=0.50, p<0.001) was greater than that between NS and BAL microbiota than that of BAL (r=0.48, p<0.001) (**Figures 4H, I**), which is consistent with the ANOSIM data based on microbial structure. In the other countries, the microbial similarities between niches had a similar pattern (**Figure S3**). Overall, the microbial connection within the respiratory tract of beef cattle existed although we observed the microbial dissimilarities among niches.

### The microaspiration in the respiratory tract of beef cattle

**Figure 5:**
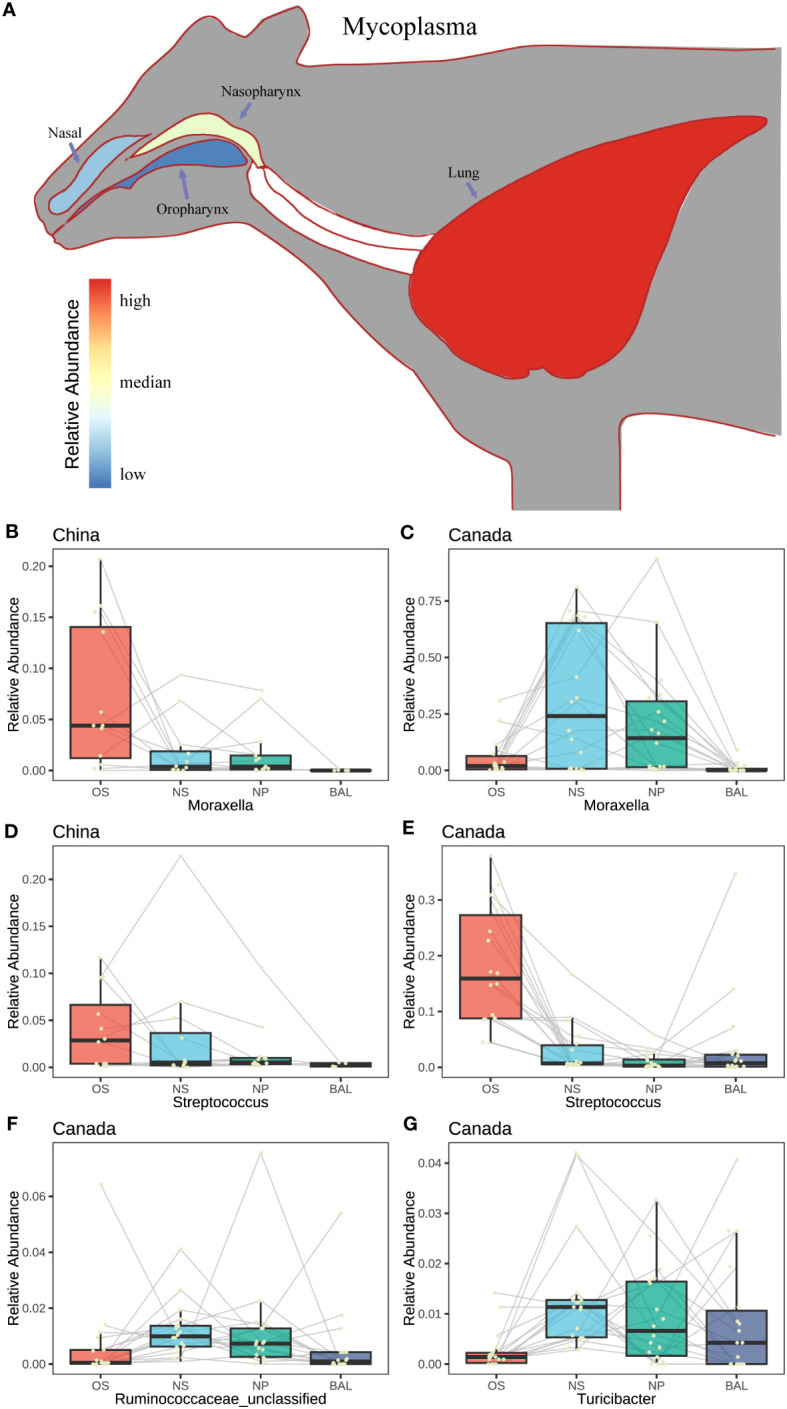
The characteristics in abundance change of the featured microbes. **(A)** Model diagram of the relative abundance change of a single bacterium (*Mycoplasma*) at different niches in the respiratory tract of animals. **(B–G)** The change of the relative abundance of the featured microbes with different niches in different countries. A yellow point represents a sample. The Connected points represent samples from the same animal.

To deeply understand the microbial connection within the respiratory tract of beef cattle, the microaspiration of the major taxa were assessed. As we observed, *Mycoplasma* associated with bovine respiratory disease existed in four sampling niches of the Canadian beef cattle and increased from the oropharynx and upper to the lung although its abundances were different (**Figure 5**). A similar pattern was also observed in the cattle from China and Italy (**Figure S4A**). In the meantime, *Streptococcus* migration among niches was the same among countries with high abundance in OS followed by NS, BAL, and NPS (**Figures 5D, E**, **S4B**). In contrast, the microaspiration of other major respiratory microbiota among countries were different. In Chinese beef cattle, *Moraxella* was higher in the oral cavity followed by NS, NPS, and lung (**Figure 5B**). However, in Canada, it was higher in NS and NPS followed by oral and lung (**Figures 5C**, **S4B**). Other important respiratory microbiota, such as *Pasteurellaceae unclassified*, *Moraxellaceae unclassified*, *Microbacteriaceae unclassified*, *Mogibacteriaceae unclassified*, *Corynebacterium*, *Pseudomonadaceae unclassified*, and *Roseburia* were abundant in the upper airway or oropharynx, but their abundance in lung varied among countries (**Figures S5**, **S6**). The microbiota commonly found in the gut showed higher abundances in NS and NPS followed by OS and BAL, such as *Ruminococcaceae unclassified* and *Turicibacter* (**Figures 5F, G**, **S4B, C**). Other gut microbiotas, including *Prevotellaceae unclassified*, *Clostridium*, *Lactobacillus*, *Lachnospiraceae unclassified*, and *Bifidobacterium*, were more enriched in the oral cavity or the upper respiratory tracts regardless of country (**Figure S7**).

### Network analysis to reveal the microbial interactions among respiratory niches

**Figure 6:**
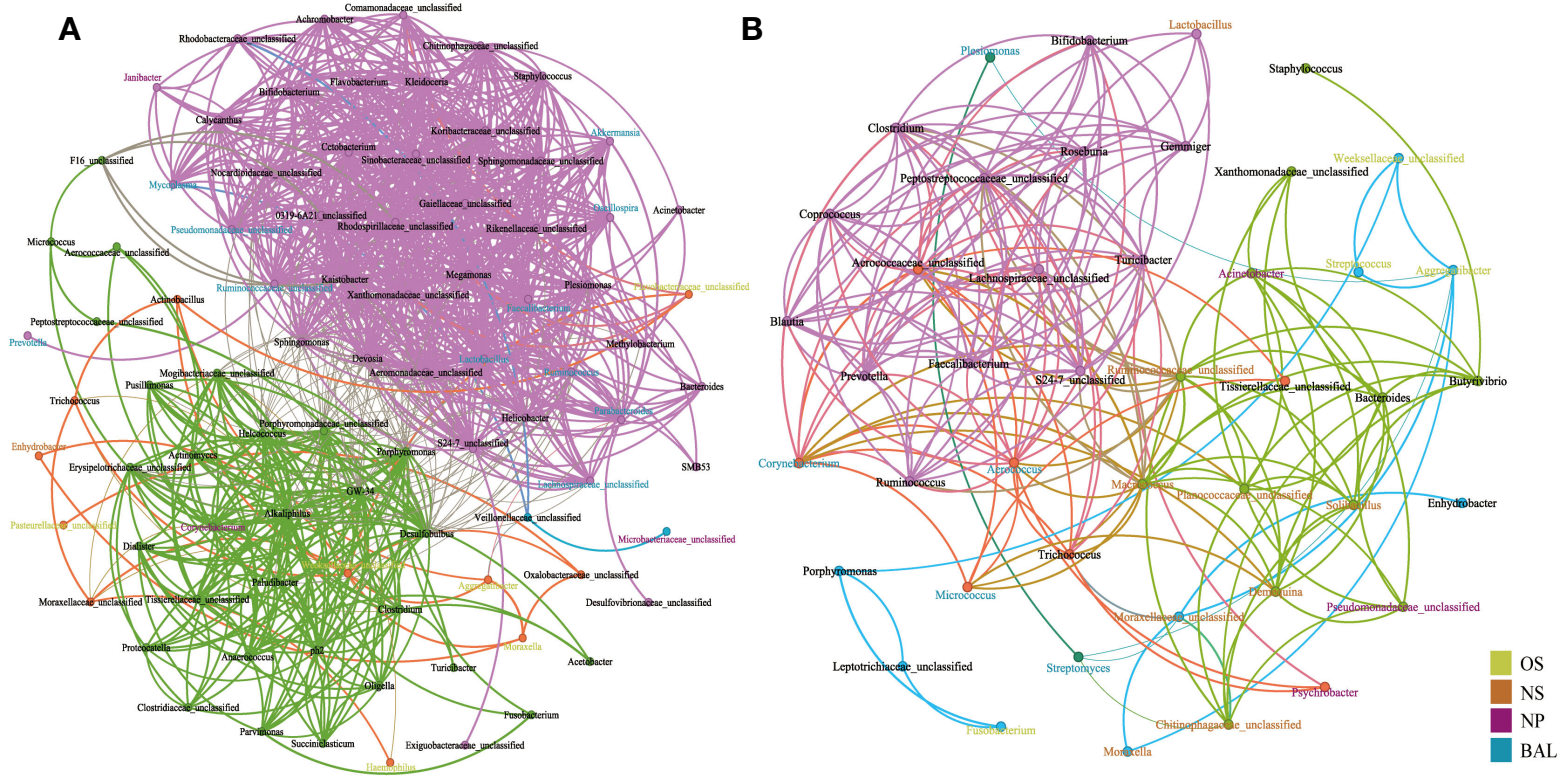
Network analysis of interactions between genus level interactions in the respiratory tract of cattle in **(A)** China and **(B)** Canada. Each node denotes a particular genus within the network and the different colors denote the featured bacteria in different niches. Each line (edge) represents a significant co-efficiency relationship (Pearson rank correlation coefficient in China > 0.5 or < −0.5 and in Canada > 0.4 or < −0.4). The network has divided all bacteria into communities of different colors through modularization. OS (Oropharynx); NS (Nasal); NP (Nasopharyngeal); BAL (Bronchoalveolar lavage).

The microbial interactions were determined using network analysis (**Figures 6**, **S8**). When using Chinese and Canadian bovine airway microbial samples, four and seven modules were observed respectively. In the meantime, more edges and nodes were found in airway samples of Chinese cattle compared to that of Canadian bovines. Interestingly, in both countries, the featured microbes within the same niche exhibited a stronger correlation in the same module. For example, in China cattle, genera for BAL identified by LEfSe that correlated with other commensal microbiotas formed a module, including *Mycoplasma*, *Lactobacillus*, *Akkermansia*, *Ruminococcaceae unclassified*, *Lachnospiraceae unclassified*, *Ruminococcus*, *Oscillospira*, and *Pseudomonadaceae unclassified* (**Figure 6**). In addition, these signature microbiotas were identified as the bridge nodes to connect different modules. The BAL signature microbiotas for the bovines from Canada, including *Corynebacterium, Aerococcus*, *Micrococcus*, were the key nodes to bright two modules. Additionally, network analysis using all four countries’ samples showed that the niches’ effects on the bacterial interaction were slight (**Figure S8**). All these results indicated that geographic effects on the bovine respiratory microbiome system were greater than niche effects, and the signature microbiota affected the microbial interactions.

## Discussion

Investigation of the respiratory microbiota in healthy bovines aid in understanding the critical roles of the microbiota in health and provide microbial insights for prevention and diagnosis of BRD. Until now, the nasopharynx has been the most popular sampling site to investigate bovine respiratory microbiota (McMullen et al., 2020a), but microbiota colonization in the nostrils and lungs is less studied and important. In this study, we found that significant differences in the respiratory microbial composition and structure of the bovine were associated with geography and sampling niches. Despite the greater variations of the upper and lower airway microbiota being observed, geographic effects on both were also identified. It is confirmed that geography influenced the physiological and biochemical environments of the bovine respiratory tract; however local physiology of niches also shaped its microbial community. The abundant bacteria for each respiratory niche were identified, and they were associated with geography. In the meantime, shared taxa among these sampling niches were found, indicating that microaspiration might exist in the bovine airway. However, the spatial connection of the bovine airway microbiota is still less studied. This study determined the spatial dynamics of the respiratory microbiota from the nostrils to the nasopharynx to the lung and found that NS and NPS microbiota were more similar compared to the lung community. The oral microbiota also showed similarities to the respiratory microbiota. In addition, the spatial connection of the bovine respiratory microbiota from worldwide geographic locations was also characterized, which allows us to understand the microaspiration of the microbiota deeply. This study elucidated the fundamental knowledge of the bovine respiratory microbiota.

Geography serving an important role in affecting the bovine respiratory microbiota has been confirmed in our previous study (Chai et al., 2022b). In this study, we also found that either the upper or lower airway microbiota in the beef cattle from China (Asia), Canada and the United States (North America), and Italy (Europe) had different diversities and composition. Calves living in worldwide geographic locations experienced environmental variations, including feed strategy, diet, altitude, temperature, etc. In this study, the dominant bacteria varied across the four countries. For instance, *Mycoplasma* was abundant in the upper and lower airway of Italy cattle but lower in other countries, and *Moraxella* was enriched in the upper airway of cattle from Canada but lower in other niches of cattle from other countries. Previously, a study found that, in Canada, the most prominently identified bacteria in the bovine nasopharynx were *Mycoplasma*, *Lactococcus*, *Moraxella*, *Histophilus*, and *Pasteurella*, while *Mannheimia*, *Mycoplasma*, *Moraxella*, *Psychrobacter*, and *Pseudomonas* were the top five genera in the nasopharynx of calves from the United States (Lima et al., 2016; McMullen et al., 2018). Thus, it’s not surprising that bovine airway microbiota from three continents showed differences. In addition, the current study first characterized the altitude effects on the bovine airway microbiota. We found that more significant differences in airway microbiota among cattle from 200 m, 500 m, 1000 m, and 1500 m masl were observed although breed and diet effects may exist. Thus, geography or external environment may have a greater influence on the bovine airway microbiota. Further studies need to deepen the environmental effects, which might benefit the probiotics production from high-altitude cattle.

Anatomical niches within the respiratory tract have different local environments (Chai et al., 2022a). Differences in microbial composition and structure in the upper and lower respiratory tract of bovine were found in the current study. The bovine nostrils had higher abundances of *Enhydrobacter, Corynebacterium, Pseudoalteromonas*, and *Phascolarctobacterium*, while the dominant genera in the nasopharynx were *Psychrobacter, Moraxella, Pseudomonadaceae unclassified, Ruminococcaceae unclassified, Roseburia, Moraxellaceae unclassified*, and *Acinetobacter*. In the lungs, we classified *Streptomyces, Plesiomonas, Turicibacter, Microbacteriaceae unclassified*, and *Mycoplasma*. Previous studies reported dominant genera in the bovine nasal cavity, such as *Psychrobacter*, *Aggregatibacter*, *Sphingomonas*, *Corynebacterium*, and *Coprococcus* (Nicola et al., 2017; McDaneld et al., 2018). The nasopharynx (the region near the caudal aspect of the nose) colonized with *Pseudomonas*, *Psychrobacter*, *Actinobacillus*, *Clostridium*, *Acinetobacter*, *Bacillus*, *Proteus*, *Bifidobacterium*, *Rathayibacter*, *Cellulomonadaceae*, *Corynebacterium*, *Jeotgalicoccus*, and *Planomicrobium* (Gaeta et al., 2017; Holman et al., 2017; Zeineldin et al., 2017a; Timsit et al., 2018; Amat et al., 2019). In the clinically healthy bovine lungs, previous studies found the genera *Mycoplasma*, *Moraxella*, *Pasteurella*, *Mannheimia*, *Bacteroides*, *Clostridium*, *Bibersteinia*, and *Prevotella* (Nicola et al., 2017; Zeineldin et al., 2017b; Klima et al., 2019). Overall, except for the variations among cattle or studies, the bacterial composition and abundances among the three popular sampling niches (nostrils, nasopharynx, and lungs) in the bovine are different but shared taxon could be observed. Investigation of the global microbial ecosystem in calves allows us to identify BRD pathogens well.

Microbial movement or dispersion within the respiratory tract is new and essential research direction as it could potentially explain the contribution of the upper airway microbiota to the lung microbiota and the respiratory health to disease (Zeineldin et al., 2019; Chai et al., 2022a). This study found that microbial communities in the nostrils and nasopharynx were correlated with those in the lungs regardless of geographic effects. One recent study concluded that nasopharyngeal microbiota may serve as the primary source for the lung microbiota in healthy calves since the nasopharyngeal region shared a similar bacterial composition with the lungs compared to other sampling niches (McMullen et al., 2020a). Similarly, bacterial overlaps between the upper and lower tracts in cattle have also been reported (Nicola et al., 2017; Zeineldin et al., 2017b; Timsit et al., 2018), indicating microbial aspiratory within the bovine respiratory tract. In healthy subjects, microbiota from the upper airway could enter the lungs via an active and continuous process in several ways, such as inhalation of air, direct mucosal dispersal, and microaspiration (Dickson et al., 2014). Furthermore, an adapted island model was applied in healthy humans, and the hypothesis is that the lung microbiome and its growth rate are more affected by microbial entry and removal processes than by the effects of the local growth environments. However, for ruminants, there is no specific statistical model to investigate the respiratory microbial movement. Thus, the current study mapped the relative abundance of the major microbiota along the bovine airway. We found that immigration of some microbiotas was similar in different countries, such as *Mycoplasma* and *Moraxella* abundant in the upper airway rather than the lungs. However, geography affecting the microaspiration of a specific bacterium was also observed. For example, the abundances of *Pasteurellaceae* and *Microbacteriaceae* were high in the upper air way but varied among countries in lung communities. Overall, the microaspiration of the bovine respiratory microbiota existed and some bacteria showed same immigration pattern no matter whatever geographies, which may help us explain the common pattern of respiratory microbiome in the world.

Oral microbiome in cattle is starting to be studied as its importance in humans has been confirmed (Dickson and Huffnagle, 2015; Dickson et al., 2016). The specific ruminating activity in cattle may cause more oral microbiota to enter into the lungs (Glendinning et al., 2017). Moreover, social grooming may cause oral bacteria to migrate into the upper airway. All these movements of microbiota are relevant to our understanding of pathogenesis in bovine health and disease (Segal and Blaser, 2014). In this study, the major bacterial signatures, including *Aggregatibacter, Streptococcus, Pasteurellaceae unclassified, Clostridium, Ruminococcus, Lactobacillus, Prevotellaceae unclassified*, and *Bifidobacterium*, were identified for the bovine oral cavity. A previous study found that *Pasteurellaceae*, *Moraxellaceae*, and *Neisseriaceae* associated with the BRD pathogens were detected in the oral cavity of calves (Barden et al., 2020). Another study found that *Pseudomonas*, *Burkholderia*, and *Actinobacteria* were the most prevalent bacteria in the mouth of healthy cattle, but *Prevotella*, *Fusobacterium*, and *Porphyromonas* were significantly increased in cattle with periodontitis (Borsanelli et al., 2018). Although the dominant species may be different, shared taxa were observed compared to these studies. Our results also found that some oral bacteria were associated with the upper and lower airway microbiota in cattle, which showed a similar pattern to a previous study (McMullen et al., 2020a). For example, *Moraxella* and *Mogibacteriaceae unclassified* were higher in both the oral cavity and nostrils. Considering cattle often lick their noses and can actually reach farther into their nostrils than other species, this is not surprising. However, the composition of the oral microbiome and its association with the bovine respiratory microbial communities is still largely unknown. The oral and oropharyngeal microbiome for health and BRD calves should be further investigated.

## Conclusions

Bacterial 16S rRNA gene sequencing demonstrated that geography affected both the upper and lower respiratory microbiota in bovine. The factors related to geography, including altitude and temperature, may contribute to microbial changes. Although the dominant genera among countries were different, shared taxa were observed, indicating that the similarities in research on bovine respiratory microbiota may elucidate the microbial roles in health and disease. Beyond the most popular sampling niche (nasopharynx) in the current studies, the microbiome in nostrils and lungs showed their specifications, which should be further investigated for better understanding of bovine respiratory disease. In addition to the effects of geography and niche on the bovine airway ecosystem, the common taxa and their immigration pattern were characterized in this study, providing some insights into bovine respiratory microbiome and health. Moreover, oral microbiota with its specific composition compared to the respiratory microbiome was associated with the nostril and lung microbial community in cattle. Therefore, all the results in this study support the notion that bacterial isolations or probiotics could be administrated into the bovine mouth or nostrils to improve lung community.

## Data availability statement

Sequences were deposited in the NCBI sequence read archive (SRA) database under Bioproject PRJNA952496, BioSamples SAMN34074578-SAMN34074618.

## Ethics statement

The animal studies were approved by Animal Ethics and Humane Animal Care of the Foshan University. The studies were conducted in accordance with the local legislation and institutional requirements. Written informed consent was obtained from the owners for the participation of their animals in this study.

## Author contributions

ZZ: Data curation, Formal Analysis, Investigation, Writing – original draft. CZ: Data curation, Investigation, Writing – original draft. YZ: Data curation, Investigation, Writing – original draft. SY: Data curation, Methodology, Resources, Writing – review & editing. FD: Data curation, Investigation, Writing – original draft. YL: Funding acquisition, Project administration, Writing – review & editing. JC: Project administration, Writing – review & editing, Conceptualization, Data curation, Formal Analysis, Investigation, Methodology, Resources, Software, Supervision, Writing – original draft.
