# Supplementary material for: The spatial dissimilarities and connections of the microbiota in the upper and lower respiratory tract of beef cattle
Source: Front Cell Infect Microbiol. 2023 Nov 6;13:1269726. doi: 10.3389/fcimb.2023.1269726 (PMC10660669; doi:10.3389/fcimb.2023.1269726)
Supplement: Supplementary file 1 [file DataSheet_1.docx]

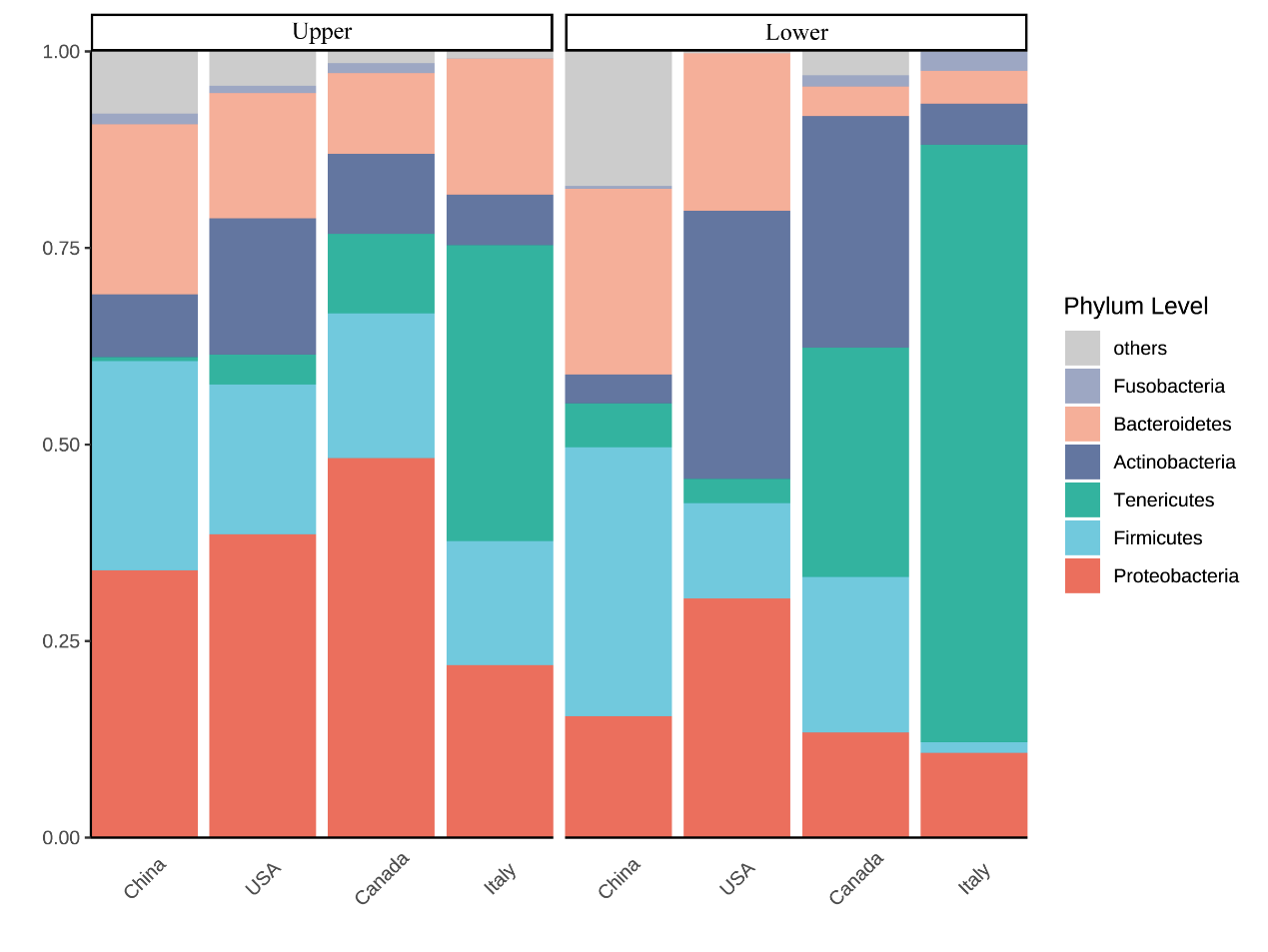


Figure S1 The composition of microorganisms with relative abundance > 1% at the phylum level. The value of the bar plot was drawn by the average value of the group.


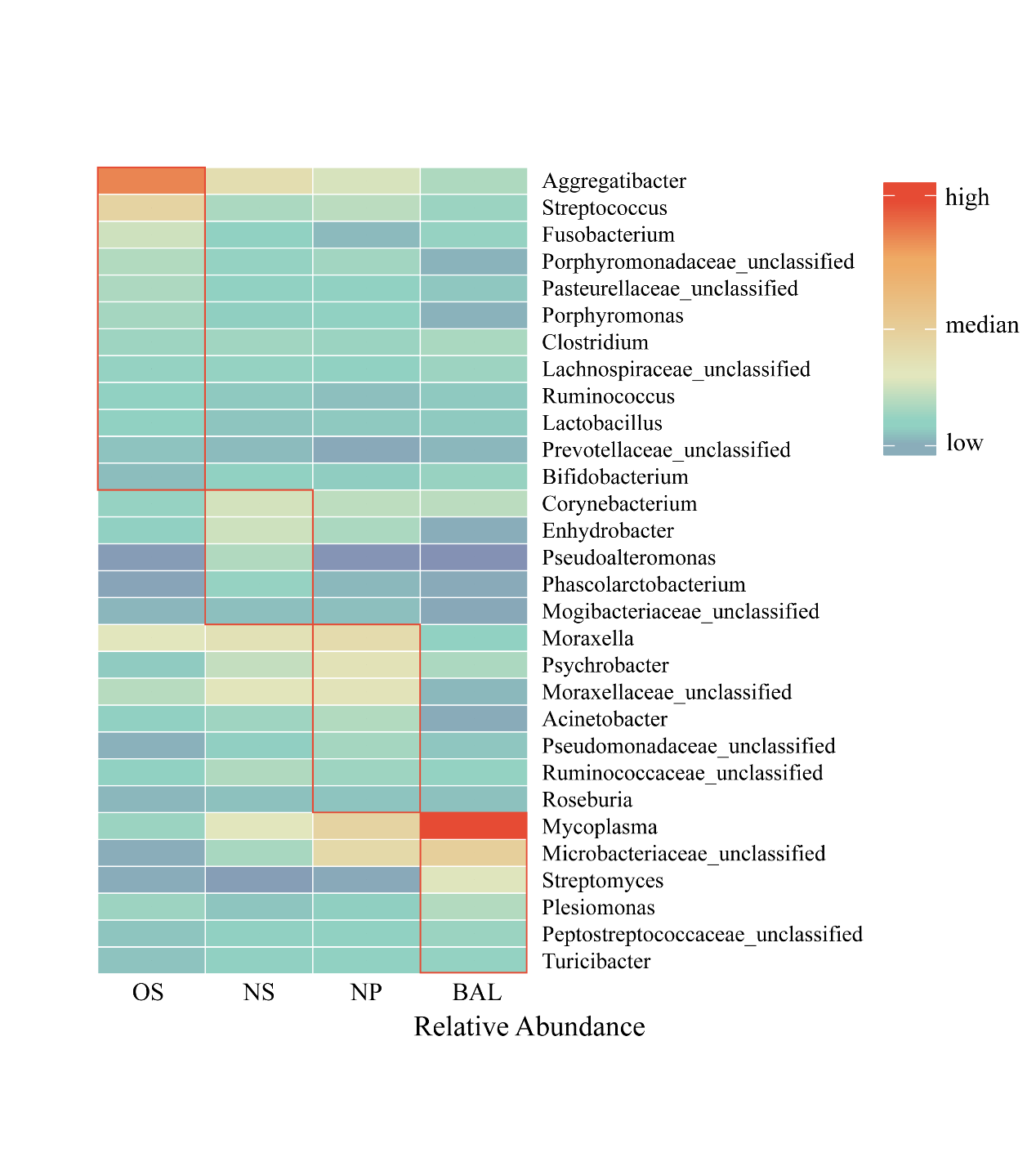


Figure S2 Abundance profile of featured microbes in different ecological niches in the respiratory tract of cattle in all samples. The average value of the relative abundance of the single bacteria were compared after making the logarithm in different niches. The featured microbes were selected based on the results of LEfSe analysis.


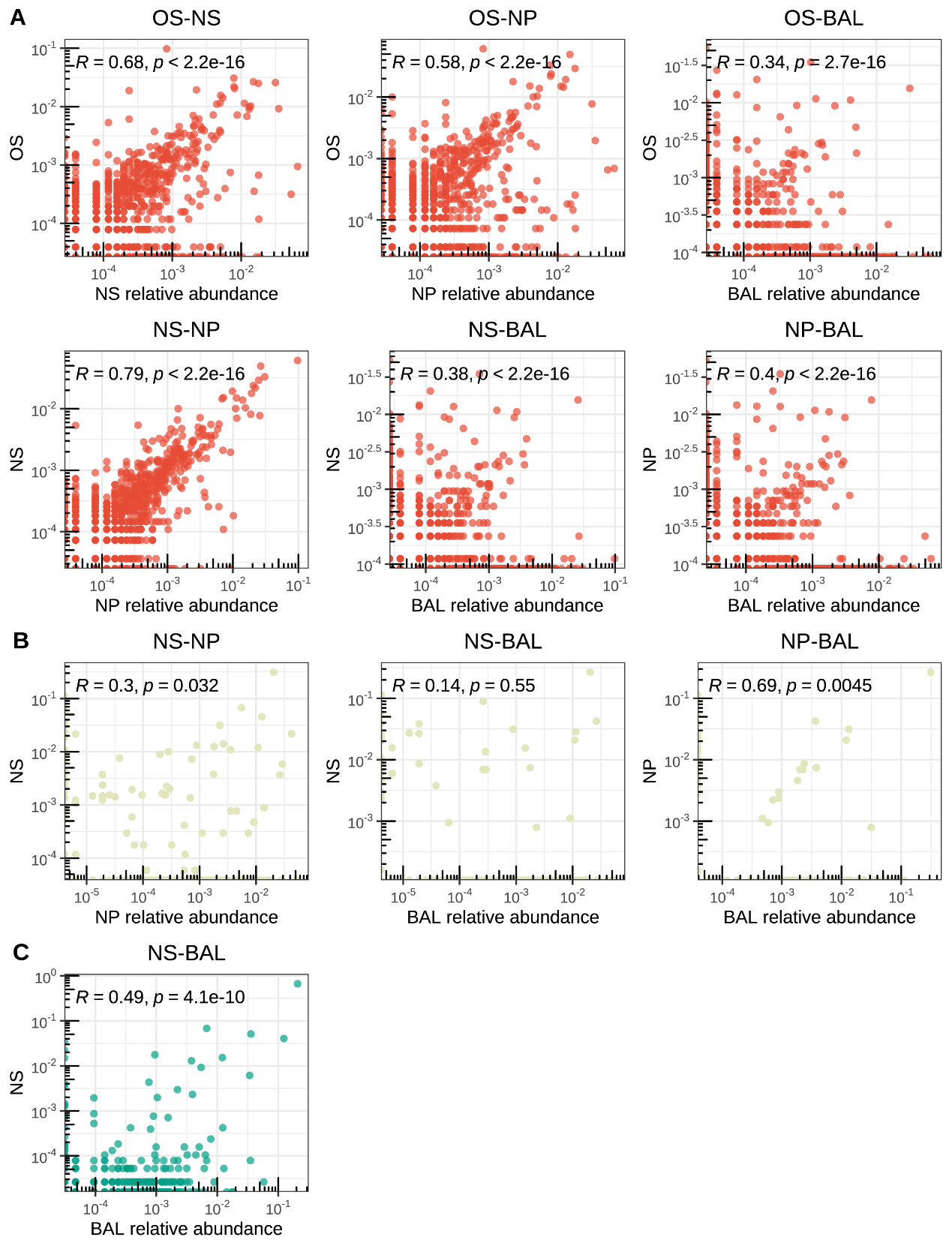


Figure S3 The correlation of the respiratory tract microbes in different niches. (A) The correlation of Chinese samples in different niches. (B) The correlation of US samples in different niches. (C) The correlation of Italian samples in different niches. Each point corresponds to the average relative abundance of a feature across all animals for each of the respiratory tract sampling niches. To measure correlation, Pearson’s r was calculated based on features abundance of two niches.


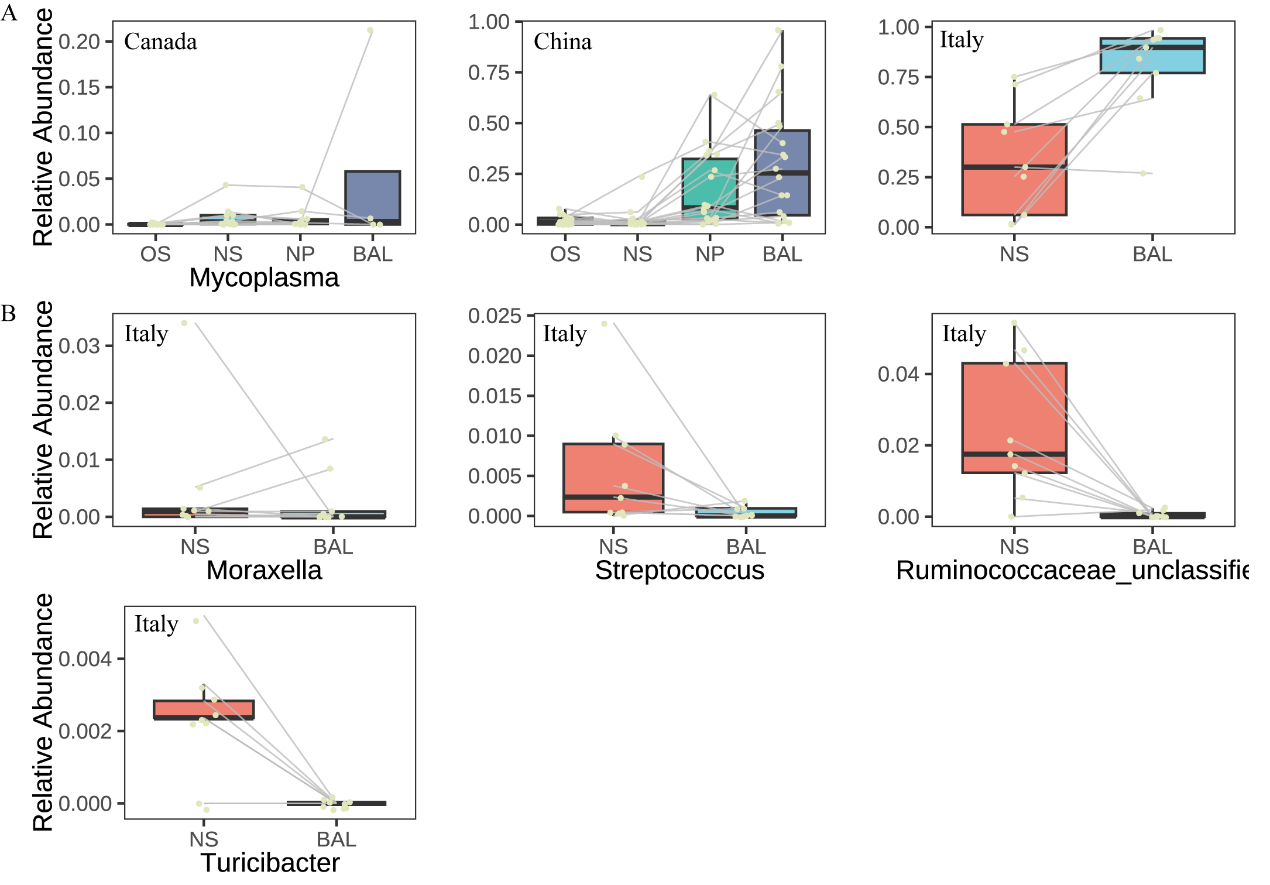


Figure S4 The characteristics in abundance change of the featured microbes. (A) Relative abundance of Mycoplasma in different niches in different countries. (B) Relative abundance of featured bacteria between NS and BAL in respiratory tract of Italian beef cattle. The Connected points represent samples from the same animal.


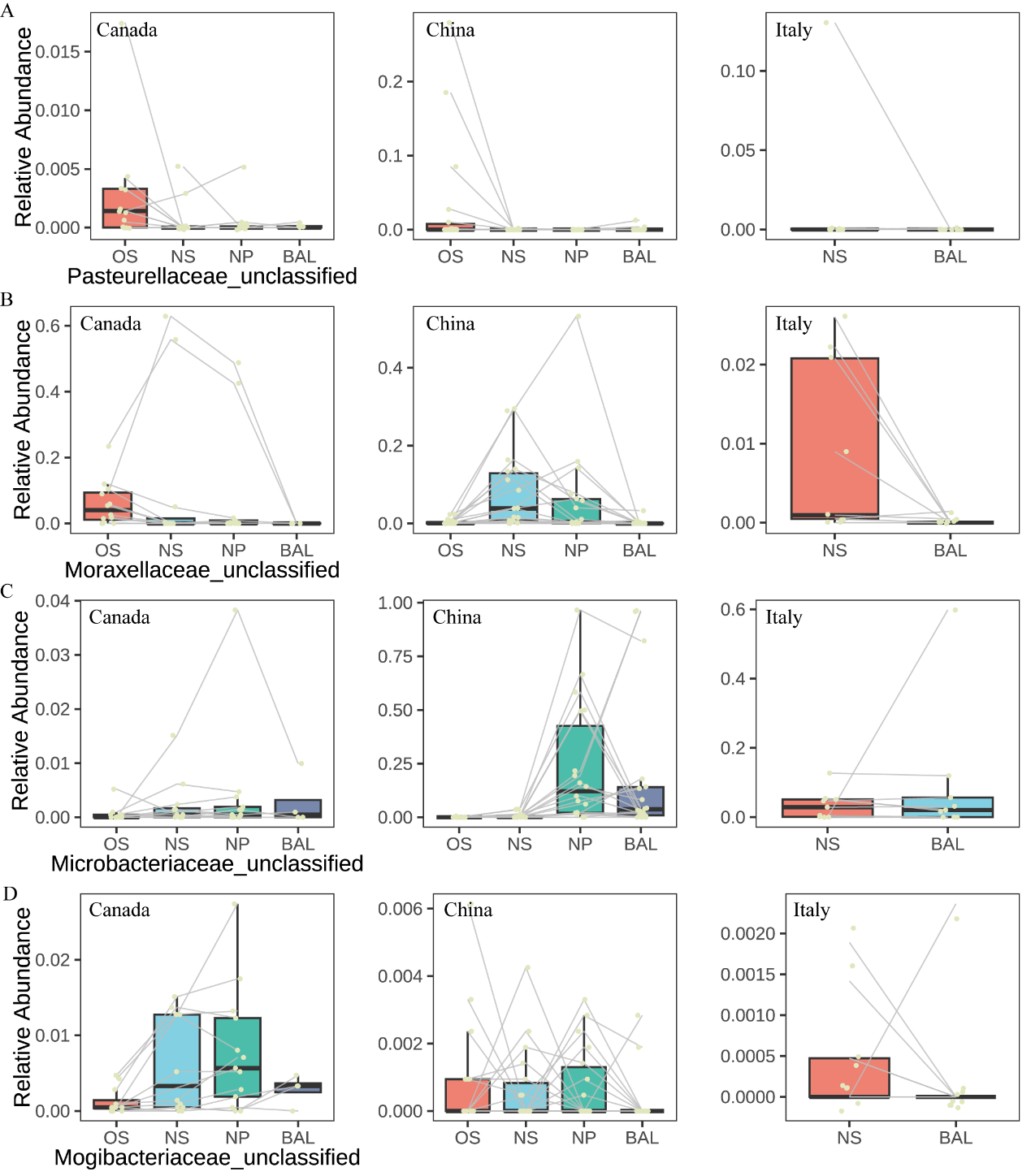


Figure S5 The characteristics in abundance change of the featured microbes. (A-D) The relative abundance of single featured microbe in different niches in different countries. The connected points represent samples from the same animal.


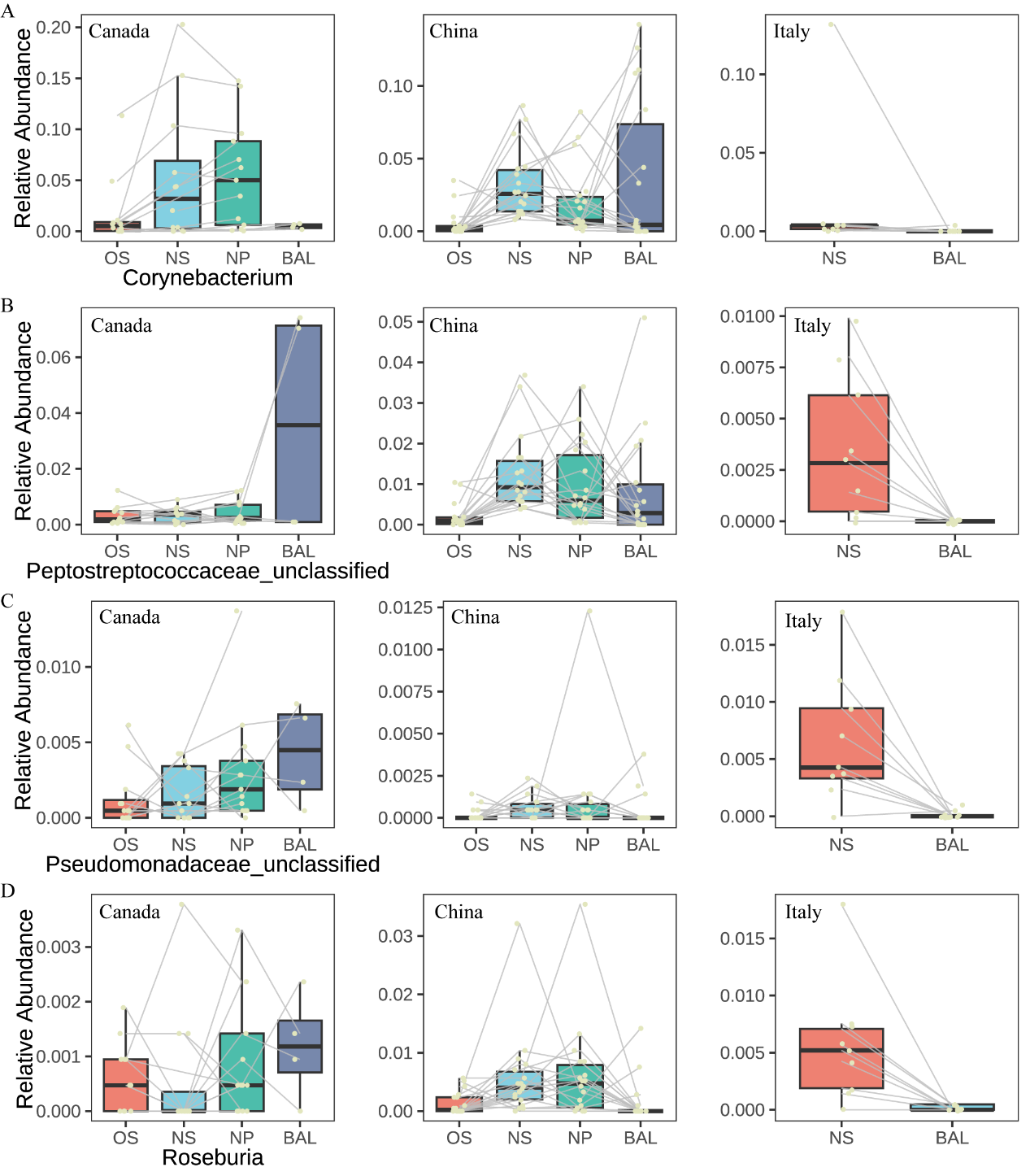


Figure S6 The characteristics in abundance change of the featured microbes. (A-D) The relative abundance of single featured microbe in different niches in different countries. The connected points represent samples from the same animal.


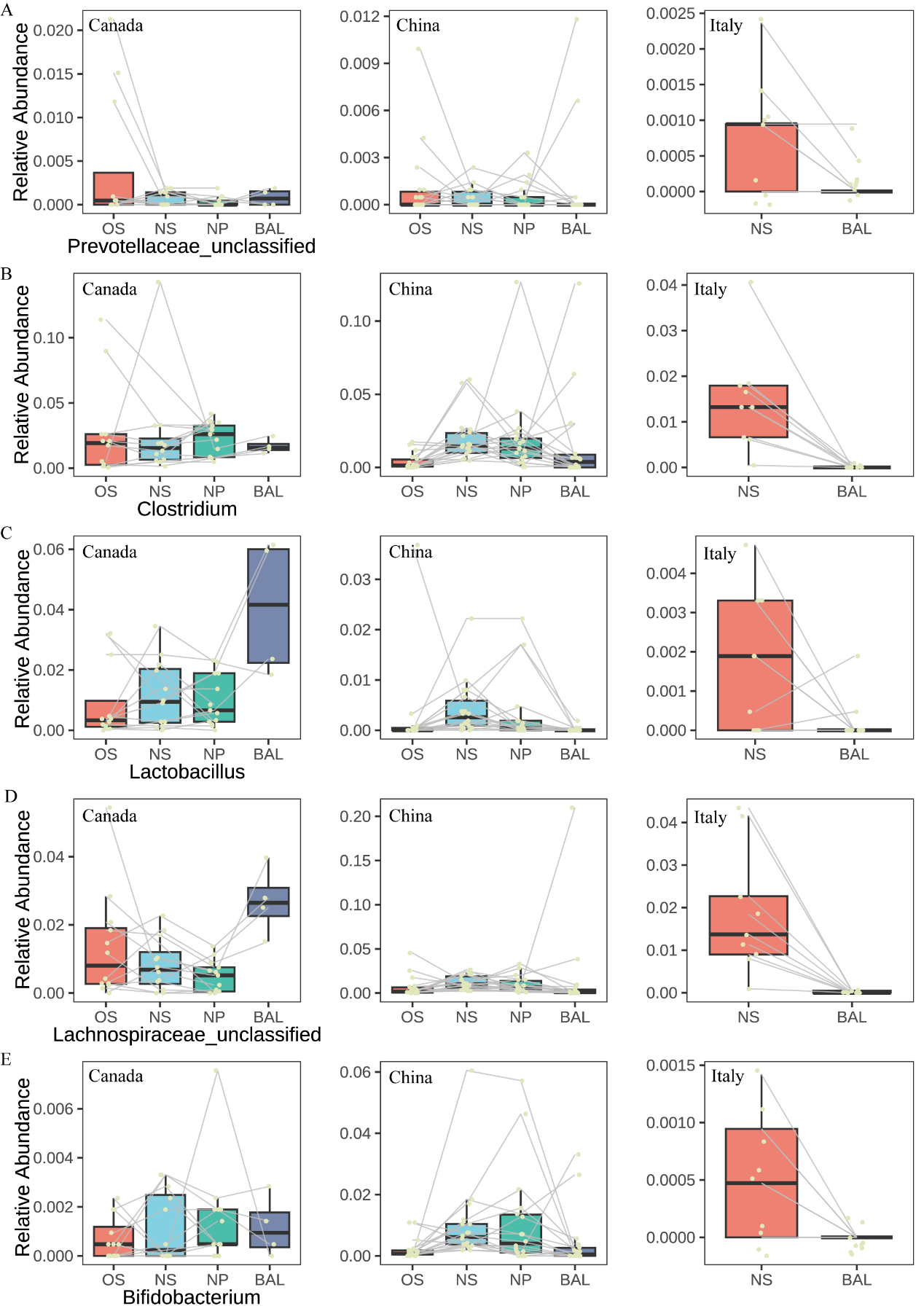


Figure S7 The characteristics in abundance change of the featured microbes. (A-E) The relative abundance of single featured microbe in different niches in different countries. The connected points represent samples from the same animal.


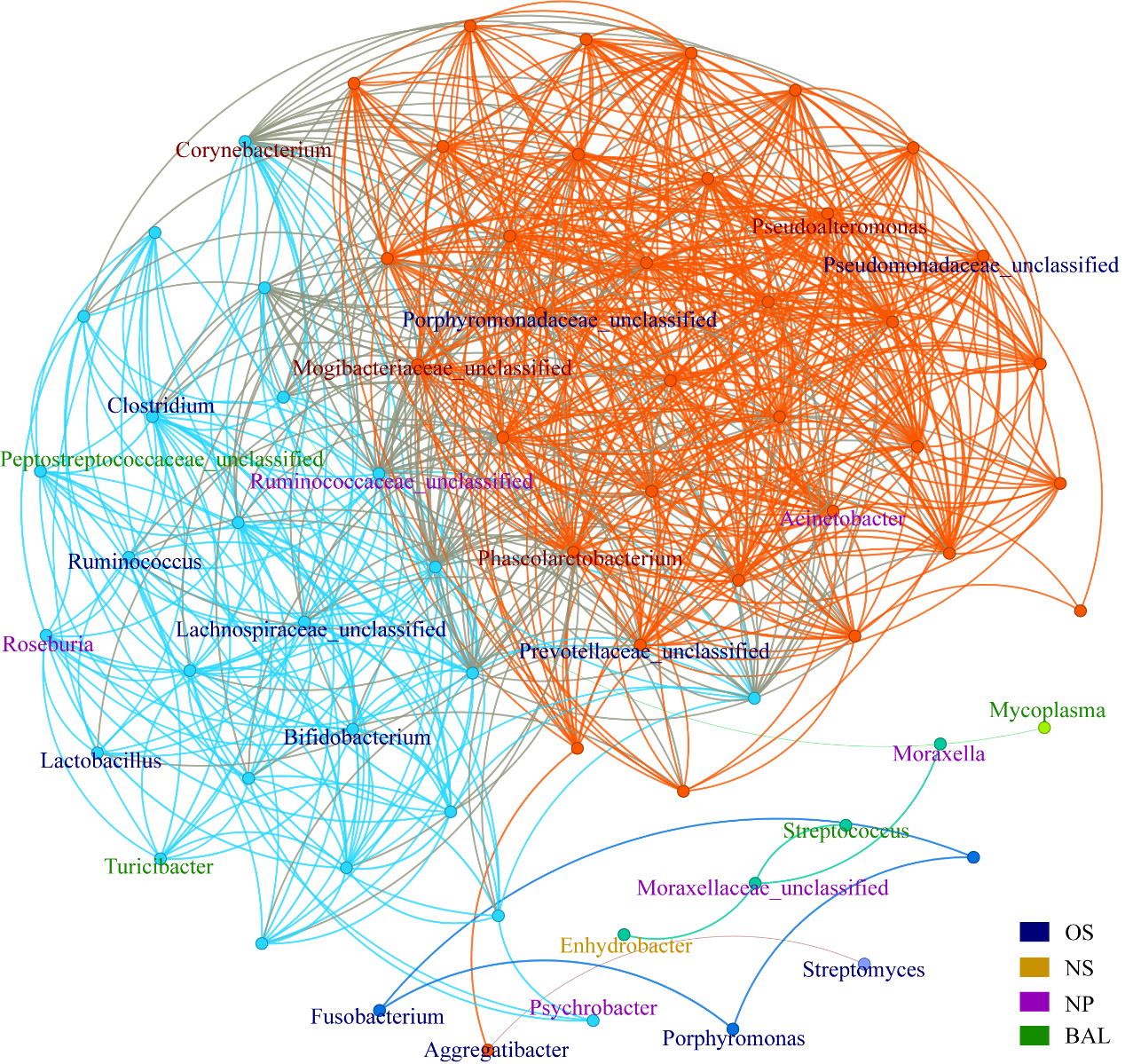


Figure S8 Network analysis of interactions between genus level interactions in the respiratory tract of cattle. Each node denotes a particular genus within the network and the different colors denote the featured bacteria in different niches. Each line (edge) represents a significant co-efficiency relationship (Pearson rank correlation coefficient > 0.4 or < −0.4). The network was divided all bacteria into communities of different colors through modularization.

Supplemental Table S1 Raw data information (PRJNA952496), two breeds from Yunnan, China. 16S rRNA v3-v4 region sequenced by Illumina Novaseq 6000.

| Accession | Breeds | Age | Sample type | Animal ID | Elevation | lat_lon |
| --- | --- | --- | --- | --- | --- | --- |
| SRR24098637 | Bos frontalis | adult | NS | F1 | 1,500m | 27.70 N 98.66 E |
| SRR24098636 | Bos frontalis | adult | NS | F2 | 1,500m | 27.70 N 98.66 E |
| SRR24098620 | Bos frontalis | adult | NS | F3 | 1,500m | 27.70 N 98.66 E |
| SRR24098609 | Bos frontalis | adult | NS | F4 | 1,500m | 27.70 N 98.66 E |
| SRR24098598 | Bos frontalis | adult | NS | F5 | 1,500m | 27.70 N 98.66 E |
| SRR24098633 | Bos frontalis | adult | NP | F1 | 1,500m | 27.70 N 98.66 E |
| SRR24098632 | Bos frontalis | adult | NP | F2 | 1,500m | 27.70 N 98.66 E |
| SRR24098631 | Bos frontalis | adult | NP | F3 | 1,500m | 27.70 N 98.66 E |
| SRR24098630 | Bos frontalis | adult | NP | F4 | 1,500m | 27.70 N 98.66 E |
| SRR24098629 | Bos frontalis | adult | NP | F5 | 1,500m | 27.70 N 98.66 E |
| SRR24098635 | Bos frontalis | adult | OS | F1 | 1,500m | 27.70 N 98.66 E |
| SRR24098634 | Bos frontalis | adult | OS | F2 | 1,500m | 27.70 N 98.66 E |
| SRR24098628 | Bos frontalis | adult | OS | F3 | 1,500m | 27.70 N 98.66 E |
| SRR24098627 | Bos frontalis | adult | OS | F4 | 1,500m | 27.70 N 98.66 E |
| SRR24098626 | Bos frontalis | adult | OS | F5 | 1,500m | 27.70 N 98.66 E |
| SRR24098625 | Bos frontalis | adult | BAL | F1 | 1,500m | 27.70 N 98.66 E |
| SRR24098624 | Bos frontalis | adult | BAL | F2 | 1,500m | 27.70 N 98.66 E |
| SRR24098623 | Bos frontalis | adult | BAL | F3 | 1,500m | 27.70 N 98.66 E |
| SRR24098622 | Bos frontalis | adult | BAL | F5 | 1,500m | 27.70 N 98.66 E |
| SRR24098621 | Bos indicus | adult | NS | YC_1 | 500m | 24.39 N 98.54 E |
| SRR24098619 | Bos indicus | adult | NS | YC_2 | 500m | 24.39 N 98.54 E |
| SRR24098618 | Bos indicus | adult | NS | YC_3 | 500m | 24.39 N 98.54 E |
| SRR24098617 | Bos indicus | adult | NS | YC_4 | 500m | 24.39 N 98.54 E |
| SRR24098616 | Bos indicus | adult | NS | YC_5 | 500m | 24.39 N 98.54 E |
| SRR24098615 | Bos indicus | adult | NS | YC_6 | 500m | 24.39 N 98.54 E |
| SRR24098614 | Bos indicus | adult | NS | YC_7 | 500m | 24.39 N 98.54 E |
| SRR24098613 | Bos indicus | adult | NP | YC_1 | 500m | 24.39 N 98.54 E |
| SRR24098612 | Bos indicus | adult | NP | YC_2 | 500m | 24.39 N 98.54 E |
| SRR24098611 | Bos indicus | adult | NP | YC_3 | 500m | 24.39 N 98.54 E |
| SRR24098610 | Bos indicus | adult | NP | YC_4 | 500m | 24.39 N 98.54 E |
| SRR24098608 | Bos indicus | adult | NP | YC_5 | 500m | 24.39 N 98.54 E |
| SRR24098607 | Bos indicus | adult | NP | YC_6 | 500m | 24.39 N 98.54 E |
| SRR24098606 | Bos indicus | adult | NP | YC_7 | 500m | 24.39 N 98.54 E |
| SRR24098605 | Bos indicus | adult | NP | YC_8 | 500m | 24.39 N 98.54 E |
| SRR24098604 | Bos indicus | adult | OS | YC_1 | 500m | 24.39 N 98.54 E |
| SRR24098603 | Bos indicus | adult | OS | YC_2 | 500m | 24.39 N 98.54 E |
| SRR24098602 | Bos indicus | adult | OS | YC_3 | 500m | 24.39 N 98.54 E |
| SRR24098601 | Bos indicus | adult | OS | YC_5 | 500m | 24.39 N 98.54 E |
| SRR24098600 | Bos indicus | adult | OS | YC_6 | 500m | 24.39 N 98.54 E |
| SRR24098599 | Bos indicus | adult | OS | YC_7 | 500m | 24.39 N 98.54 E |
| SRR24098597 | Bos indicus | adult | OS | YC_8 | 500m | 24.39 N 98.54 E |

Supplemental Table S2 Summary of a publicly available dataset of bacterial 16S rRNA genes of the bovine respiratory microbiota.

| Study title | Country of origin | Sample type | Cattle breed | Total number of samples | Sequencing platform and hypervariable regions | Data availability accession number |
| --- | --- | --- | --- | --- | --- | --- |
| Relationship between nasopharyngeal and bronchoalveolar microbial communities in clinically healthy feedlot cattle | USA | NP and BAL | Charolais | 16 | Illumina MiSeq (V3-V4) | PRJNA323521 |
| Antibiotic treatment in feedlot cattle: a longitudinal study of the effect of oxytetracycline and tulathromycin on the fecal and nasopharyngeal microbiota | Canada | NS | Angus-Herford | 36 | Illumina MiSeq (V4) | PRJNA423104 |
| Injectable antimicrobials in commercial feedlot cattle and their effect on the nasopharyngeal microbiota and antimicrobial resistance | Canada | NP | Angus-Herford | 30 | Illumina MiSeq (V4) | PRJNA394129 |
| The nasopharyngeal microbiota of beef cattle before and after transport to a feedlot | Canada | NP | Angus-Herford | 13 | Illumina MiSeq (V4) | PRJNA296393 |
| Topography of the respiratory tract bacterial microbiota in cattle | Canada | OS, NS, NP and BAL | Angus-Herford | 198 | Illumina MiSeq (V3-V4) | PRJNA596300 |
| Identification of bovine respiratory disease through the nasal microbiome | USA | NS | Holstein | 75 | Illumina MiSeq (V4) | PRJNA746809 |
| Characterization of the upper and lower respiratory tract microbiota in Piedmontese calves | Italy | NS and BAL | Piedmontese | 32 | Illumina MiSeq (V3-V4) | PRJNA383722 |
| This study | China | OS, NS, NP and BAL | Bos frontalis and Bos indicus | 41 | Illumina Novaseq 6000 (V3-V4) | PRJNA952496 |

Supplemental Table S3 The quality control output of the 16S rawdata

| sample ID | Niches | Country | total-input-reads | after-quality-filtered-reads | after-deblur-feature-count |
| --- | --- | --- | --- | --- | --- |
| 1-SRR5461564 | NS | Italy | 5523 | 811 | 262 |
| 1-SRR5461565 | NS | Italy | 30732 | 6592 | 2553 |
| 1-SRR5461566 | BAL | Italy | 12532 | 1181 | 505 |
| 1-SRR5461567 | BAL | Italy | 137162 | 29002 | 16778 |
| 1-SRR5461568 | NS | Italy | 81463 | 80357 | 65555 |
| 1-SRR5461569 | BAL | Italy | 88760 | 20065 | 10598 |
| 1-SRR5461570 | BAL | Italy | 44087 | 6601 | 3348 |
| 1-SRR5461571 | NS | Italy | 1682 | 1637 | 1052 |
| 1-SRR5461572 | NS | Italy | 98373 | 97901 | 70600 |
| 1-SRR5461573 | NS | Italy | 11485 | 11393 | 8408 |
| 1-SRR5461574 | BAL | Italy | 36607 | 36252 | 29696 |
| 1-SRR5461575 | BAL | Italy | 83942 | 83272 | 71428 |
| 1-SRR5461576 | BAL | Italy | 62374 | 61585 | 51504 |
| 1-SRR5461577 | BAL | Italy | 26269 | 25983 | 21915 |
| 1-SRR5461578 | BAL | Italy | 32630 | 32071 | 26236 |
| 1-SRR5461579 | BAL | Italy | 30499 | 30135 | 23885 |
| 1-SRR5461580 | BAL | Italy | 30911 | 30499 | 25064 |
| 1-SRR5461581 | BAL | Italy | 51740 | 51136 | 43818 |
| 1-SRR5461582 | BAL | Italy | 21228 | 21117 | 18072 |
| 1-SRR5461583 | BAL | Italy | 89857 | 88990 | 76422 |
| 1-SRR5461584 | NS | Italy | 7517 | 7456 | 5834 |
| 1-SRR5461585 | NS | Italy | 12827 | 12713 | 9322 |
| 1-SRR5461586 | NS | Italy | 2639 | 2600 | 1879 |
| 1-SRR5461587 | NS | Italy | 24765 | 24530 | 18202 |
| 1-SRR5461588 | BAL | Italy | 5755 | 5703 | 4782 |
| 1-SRR5461589 | BAL | Italy | 10677 | 10545 | 8494 |
| 1-SRR5461590 | NS | Italy | 20790 | 20659 | 16859 |
| 1-SRR5461591 | NS | Italy | 15451 | 15330 | 11787 |
| 1-SRR5461592 | NS | Italy | 19690 | 19534 | 15165 |
| 1-SRR5461593 | BAL | Italy | 49619 | 49289 | 42280 |
| 1-SRR5461594 | BAL | Italy | 88905 | 88305 | 75860 |
| 1-SRR5461595 | BAL | Italy | 89020 | 88101 | 71030 |
| 2-SRR3650980 | NP | USA | 3474 | 3474 | 2199 |
| 2-SRR3650981 | BAL | USA | 5385 | 5385 | 3410 |
| 2-SRR3650982 | NP | USA | 3828 | 3828 | 2191 |
| 2-SRR3650983 | NP | USA | 3643 | 3643 | 2308 |
| 2-SRR3650984 | NP | USA | 15796 | 15796 | 10150 |
| 2-SRR3650985 | BAL | USA | 408 | 408 | 268 |
| 2-SRR3650986 | BAL | USA | 167 | 167 | 106 |
| 2-SRR3650987 | NP | USA | 19102 | 19102 | 11893 |
| 2-SRR3650988 | NP | USA | 3519 | 3519 | 2318 |
| 2-SRR3650989 | BAL | USA | 3086 | 3086 | 1992 |
| 2-SRR3651005 | NP | USA | 16588 | 16588 | 10212 |
| 2-SRR3651006 | BAL | USA | 17029 | 17029 | 9678 |
| 2-SRR3651007 | BAL | USA | 443 | 443 | 315 |
| 2-SRR3651008 | NP | USA | 26086 | 26086 | 15679 |
| 2-SRR3651009 | BAL | USA | 237 | 237 | 129 |
| 2-SRR3653415 | BAL | USA | 15796 | 15796 | 10150 |
| 4-SRR6446921 | NS | Canada | 19370 | 19370 | 11800 |
| 4-SRR6446923 | NS | Canada | 47303 | 47303 | 22606 |
| 4-SRR6446924 | NS | Canada | 31173 | 31173 | 18247 |
| 4-SRR6446959 | NS | Canada | 18922 | 18922 | 10599 |
| 4-SRR6446986 | NS | Canada | 11176 | 11176 | 6116 |
| 4-SRR6447024 | NS | Canada | 24866 | 24866 | 15777 |
| 4-SRR6447028 | NS | Canada | 22538 | 22538 | 14228 |
| 4-SRR6447029 | NS | Canada | 18948 | 18948 | 10344 |
| 4-SRR6447033 | NS | Canada | 20281 | 20281 | 12051 |
| 4-SRR6447064 | NS | Canada | 35516 | 35516 | 22336 |
| 4-SRR6447066 | NS | Canada | 32450 | 32450 | 20376 |
| 4-SRR6447068 | NS | Canada | 40330 | 40330 | 25506 |
| 4-SRR6447071 | NS | Canada | 35321 | 35321 | 22025 |
| 4-SRR6447104 | NS | Canada | 14067 | 14067 | 7959 |
| 4-SRR6447150 | NS | Canada | 27688 | 27688 | 17231 |
| 4-SRR6447152 | NS | Canada | 15782 | 15782 | 9026 |
| 4-SRR6447154 | NS | Canada | 23654 | 23654 | 13666 |
| 4-SRR6447156 | NS | Canada | 38484 | 38484 | 23492 |
| 4-SRR6447158 | NS | Canada | 19523 | 19523 | 10990 |
| 4-SRR6447224 | NS | Canada | 31209 | 31209 | 15910 |
| 4-SRR6447277 | NS | Canada | 32244 | 32244 | 17858 |
| 4-SRR6447279 | NS | Canada | 15101 | 15101 | 8483 |
| 4-SRR6447288 | NS | Canada | 15185 | 15185 | 8761 |
| 4-SRR6447290 | NS | Canada | 18632 | 18632 | 11005 |
| 4-SRR6447292 | NS | Canada | 23647 | 23647 | 13725 |
| 4-SRR6447294 | NS | Canada | 22169 | 22169 | 12816 |
| 4-SRR6447296 | NS | Canada | 24216 | 24216 | 14827 |
| 4-SRR6447313 | NS | Canada | 24249 | 24249 | 14754 |
| 4-SRR6447315 | NS | Canada | 26500 | 26500 | 16363 |
| 4-SRR6447317 | NS | Canada | 27493 | 27493 | 15293 |
| 4-SRR6447318 | NS | Canada | 28485 | 28485 | 17335 |
| 4-SRR6447320 | NS | Canada | 23887 | 23887 | 13487 |
| 4-SRR6447355 | NS | Canada | 10377 | 10377 | 6002 |
| 4-SRR6447370 | NS | Canada | 22797 | 22797 | 12011 |
| 4-SRR6447380 | NS | Canada | 21140 | 21140 | 11966 |
| 4-SRR6447394 | NS | Canada | 13617 | 13617 | 8126 |
| 5-SRR5823805 | NP | Canada | 40330 | 40330 | 20735 |
| 5-SRR5823807 | NP | Canada | 41466 | 41466 | 22824 |
| 5-SRR5823808 | NP | Canada | 38268 | 38268 | 21495 |
| 5-SRR5823810 | NP | Canada | 37611 | 37611 | 22225 |
| 5-SRR5823811 | NP | Canada | 33350 | 33350 | 19158 |
| 5-SRR5823812 | NP | Canada | 24902 | 24902 | 14411 |
| 5-SRR5823813 | NP | Canada | 33460 | 33460 | 18530 |
| 5-SRR5823814 | NP | Canada | 33346 | 33346 | 17885 |
| 5-SRR5823816 | NP | Canada | 35199 | 35199 | 16831 |
| 5-SRR5823817 | NP | Canada | 33092 | 33092 | 18678 |
| 5-SRR5823821 | NP | Canada | 38900 | 38900 | 24490 |
| 5-SRR5823825 | NP | Canada | 30402 | 30402 | 16055 |
| 5-SRR5823828 | NP | Canada | 34286 | 34286 | 19909 |
| 5-SRR5823829 | NP | Canada | 33445 | 33445 | 21162 |
| 5-SRR5823830 | NP | Canada | 40996 | 40996 | 24241 |
| 5-SRR5823831 | NP | Canada | 35258 | 35258 | 18891 |
| 5-SRR5823841 | NP | Canada | 38796 | 38796 | 21498 |
| 5-SRR5823843 | NP | Canada | 29213 | 29213 | 19436 |
| 5-SRR5823844 | NP | Canada | 35019 | 35019 | 19319 |
| 5-SRR5823847 | NP | Canada | 37414 | 37414 | 23579 |
| 5-SRR5823851 | NP | Canada | 32778 | 32778 | 18931 |
| 5-SRR5823852 | NP | Canada | 37409 | 37409 | 21527 |
| 5-SRR5823853 | NP | Canada | 39343 | 39343 | 21063 |
| 5-SRR5823854 | NP | Canada | 44596 | 44596 | 24769 |
| 5-SRR5823855 | NP | Canada | 35277 | 35277 | 20093 |
| 5-SRR5823856 | NP | Canada | 36198 | 36198 | 22267 |
| 5-SRR5823857 | NP | Canada | 37192 | 37192 | 20489 |
| 5-SRR5823858 | NP | Canada | 38629 | 38629 | 21321 |
| 5-SRR5823859 | NP | Canada | 48507 | 48507 | 25839 |
| 5-SRR5823860 | NP | Canada | 29169 | 29169 | 16599 |
| 6-SRR2532218 | NP | Canada | 27191 | 27191 | 12256 |
| 6-SRR2532222 | NP | Canada | 66510 | 66510 | 31582 |
| 6-SRR2532271 | NP | Canada | 57783 | 57783 | 26542 |
| 6-SRR2532293 | NP | Canada | 64718 | 64718 | 34947 |
| 6-SRR2532297 | NP | Canada | 30726 | 30726 | 14050 |
| 6-SRR2532314 | NP | Canada | 43085 | 43085 | 20822 |
| 6-SRR2532400 | NP | Canada | 60625 | 60625 | 28447 |
| 6-SRR2532408 | NP | Canada | 52792 | 52792 | 25781 |
| 6-SRR2532412 | NP | Canada | 41598 | 41598 | 18239 |
| 6-SRR2532416 | NP | Canada | 40835 | 40835 | 18360 |
| 6-SRR2532417 | NP | Canada | 61529 | 61529 | 33762 |
| 6-SRR2532430 | NP | Canada | 51978 | 51978 | 23744 |
| 6-SRR2532435 | NP | Canada | 24444 | 24444 | 11265 |
| 8-SRR10739586 | BAL | Canada | 332 | 332 | 206 |
| 8-SRR10739587 | OS | Canada | 83425 | 83425 | 44824 |
| 8-SRR10739588 | BAL | Canada | 71365 | 71365 | 45656 |
| 8-SRR10739590 | BAL | Canada | 38300 | 38300 | 23576 |
| 8-SRR10739591 | NP | Canada | 80843 | 80843 | 52513 |
| 8-SRR10739592 | BAL | Canada | 21018 | 21018 | 13064 |
| 8-SRR10739593 | OS | Canada | 82986 | 82986 | 45342 |
| 8-SRR10739594 | BAL | Canada | 30500 | 30500 | 18955 |
| 8-SRR10739597 | NS | Canada | 85919 | 85919 | 52228 |
| 8-SRR10739600 | BAL | Canada | 17743 | 17743 | 11002 |
| 8-SRR10739601 | BAL | Canada | 6482 | 6482 | 3917 |
| 8-SRR10739602 | BAL | Canada | 18226 | 18226 | 11610 |
| 8-SRR10739604 | BAL | Canada | 45975 | 45975 | 27862 |
| 8-SRR10739607 | NP | Canada | 80193 | 80193 | 49839 |
| 8-SRR10739608 | BAL | Canada | 54284 | 54284 | 34955 |
| 8-SRR10739609 | BAL | Canada | 27093 | 27093 | 16966 |
| 8-SRR10739610 | NP | Canada | 68559 | 68559 | 40556 |
| 8-SRR10739611 | BAL | Canada | 3360 | 3360 | 2165 |
| 8-SRR10739612 | BAL | Canada | 55467 | 55467 | 34669 |
| 8-SRR10739613 | BAL | Canada | 30403 | 30403 | 19108 |
| 8-SRR10739615 | BAL | Canada | 36463 | 36463 | 23145 |
| 8-SRR10739616 | BAL | Canada | 55579 | 55579 | 35293 |
| 8-SRR10739617 | BAL | Canada | 50353 | 50353 | 32666 |
| 8-SRR10739619 | BAL | Canada | 40277 | 40277 | 26702 |
| 8-SRR10739620 | NS | Canada | 82527 | 82527 | 54310 |
| 8-SRR10739624 | OS | Canada | 77273 | 77273 | 40064 |
| 8-SRR10739629 | BAL | Canada | 48889 | 48889 | 30723 |
| 8-SRR10739630 | BAL | Canada | 83154 | 83154 | 53921 |
| 8-SRR10739634 | BAL | Canada | 39853 | 39853 | 25624 |
| 8-SRR10739636 | NP | Canada | 72010 | 72010 | 44204 |
| 8-SRR10739637 | OS | Canada | 88760 | 88760 | 52276 |
| 8-SRR10739638 | BAL | Canada | 35279 | 35279 | 22600 |
| 8-SRR10739639 | NS | Canada | 90691 | 90691 | 53454 |
| 8-SRR10739641 | BAL | Canada | 3566 | 3566 | 2188 |
| 8-SRR10739642 | NP | Canada | 90395 | 90395 | 57054 |
| 8-SRR10739644 | NS | Canada | 84217 | 84217 | 46686 |
| 8-SRR10739647 | BAL | Canada | 2116 | 2116 | 1427 |
| 8-SRR10739650 | BAL | Canada | 13553 | 13553 | 8761 |
| 8-SRR10739651 | BAL | Canada | 33624 | 33624 | 21170 |
| 8-SRR10739653 | NP | Canada | 76384 | 76384 | 49695 |
| 8-SRR10739654 | BAL | Canada | 88495 | 88495 | 60753 |
| 8-SRR10739655 | BAL | Canada | 41275 | 41275 | 25245 |
| 8-SRR10739657 | NP | Canada | 73441 | 73441 | 48250 |
| 8-SRR10739659 | NP | Canada | 59799 | 59799 | 37488 |
| 8-SRR10739662 | BAL | Canada | 24825 | 24825 | 15978 |
| 8-SRR10739663 | NS | Canada | 79153 | 79153 | 47380 |
| 8-SRR10739665 | OS | Canada | 76551 | 76551 | 41009 |
| 8-SRR10739666 | BAL | Canada | 32251 | 32251 | 20615 |
| 8-SRR10739667 | BAL | Canada | 10115 | 10115 | 6496 |
| 8-SRR10739668 | NS | Canada | 81081 | 81081 | 45869 |
| 8-SRR10739672 | NS | Canada | 91253 | 91253 | 58521 |
| 8-SRR10739673 | NS | Canada | 64481 | 64481 | 41799 |
| 8-SRR10739674 | BAL | Canada | 17848 | 17848 | 11438 |
| 8-SRR10739676 | NP | Canada | 68618 | 68618 | 47241 |
| 8-SRR10739677 | BAL | Canada | 24103 | 24103 | 14670 |
| 8-SRR10739679 | NP | Canada | 46093 | 46093 | 30360 |
| 8-SRR10739681 | BAL | Canada | 79248 | 79248 | 55466 |
| 8-SRR10739685 | BAL | Canada | 22310 | 22310 | 15119 |
| 8-SRR10739687 | BAL | Canada | 3893 | 3893 | 2602 |
| 8-SRR10739689 | NP | Canada | 82163 | 82163 | 53423 |
| 8-SRR10739690 | BAL | Canada | 83808 | 83808 | 57973 |
| 8-SRR10739691 | BAL | Canada | 52218 | 52218 | 33259 |
| 8-SRR10739692 | NP | Canada | 63423 | 63423 | 41286 |
| 8-SRR10739693 | BAL | Canada | 27010 | 27010 | 17924 |
| 8-SRR10739694 | BAL | Canada | 68987 | 68987 | 45812 |
| 8-SRR10739695 | NP | Canada | 68919 | 68919 | 46785 |
| 8-SRR10739696 | BAL | Canada | 13950 | 13950 | 9727 |
| 8-SRR10739699 | BAL | Canada | 23545 | 23545 | 14994 |
| 8-SRR10739701 | BAL | Canada | 48498 | 48498 | 31679 |
| 8-SRR10739704 | OS | Canada | 75820 | 75820 | 45566 |
| 8-SRR10739705 | BAL | Canada | 20824 | 20824 | 13684 |
| 8-SRR10739707 | NS | Canada | 68016 | 68016 | 38895 |
| 8-SRR10739708 | BAL | Canada | 25508 | 25508 | 16714 |
| 8-SRR10739710 | NS | Canada | 74710 | 74710 | 43868 |
| 8-SRR10739711 | NS | Canada | 100868 | 100868 | 62275 |
| 8-SRR10739713 | BAL | Canada | 2987 | 2987 | 1976 |
| 8-SRR10739714 | NS | Canada | 34444 | 34444 | 22676 |
| 8-SRR10739715 | BAL | Canada | 9819 | 9819 | 6322 |
| 8-SRR10739718 | NS | Canada | 72122 | 72122 | 45050 |
| 8-SRR10739719 | BAL | Canada | 18345 | 18345 | 12040 |
| 8-SRR10739720 | BAL | Canada | 12260 | 12260 | 8235 |
| 8-SRR10739723 | BAL | Canada | 19438 | 19438 | 12231 |
| 8-SRR10739729 | BAL | Canada | 5333 | 5333 | 3614 |
| 8-SRR10739732 | BAL | Canada | 48274 | 48274 | 31427 |
| 8-SRR10739733 | BAL | Canada | 34035 | 34035 | 21112 |
| 8-SRR10739734 | NS | Canada | 83730 | 83730 | 55359 |
| 8-SRR10739735 | NP | Canada | 55982 | 55982 | 37991 |
| 8-SRR10739736 | BAL | Canada | 15279 | 15279 | 9893 |
| 8-SRR10739737 | BAL | Canada | 8947 | 8947 | 5961 |
| 8-SRR10739741 | NP | Canada | 57490 | 57490 | 38120 |
| 8-SRR10739743 | NS | Canada | 58738 | 58738 | 37614 |
| 8-SRR10739748 | BAL | Canada | 54440 | 54440 | 37183 |
| 8-SRR10739750 | NS | Canada | 72753 | 72753 | 45511 |
| 8-SRR10739751 | BAL | Canada | 21905 | 21905 | 14393 |
| 8-SRR10739754 | BAL | Canada | 19747 | 19747 | 13505 |
| 8-SRR10739755 | BAL | Canada | 3846 | 3846 | 2308 |
| 8-SRR10739756 | NP | Canada | 74761 | 74761 | 50165 |
| 8-SRR10739757 | NP | Canada | 69142 | 69142 | 47889 |
| 8-SRR10739759 | OS | Canada | 75669 | 75669 | 42586 |
| 8-SRR10739760 | BAL | Canada | 26669 | 26669 | 17781 |
| 8-SRR10739762 | BAL | Canada | 8425 | 8425 | 5585 |
| 8-SRR10739763 | NS | Canada | 67025 | 67025 | 44364 |
| 8-SRR10739764 | NS | Canada | 67849 | 67849 | 48185 |
| 8-SRR10739765 | NS | Canada | 81576 | 81576 | 51958 |
| 8-SRR10739766 | BAL | Canada | 88705 | 88705 | 58586 |
| 8-SRR10739767 | NP | Canada | 105316 | 105316 | 67355 |
| 8-SRR10739769 | BAL | Canada | 88224 | 88224 | 60761 |
| 8-SRR10739770 | NS | Canada | 61286 | 61286 | 38817 |
| 8-SRR10739771 | BAL | Canada | 3941 | 3941 | 2766 |
| 8-SRR10739773 | BAL | Canada | 13627 | 13627 | 9163 |
| 8-SRR10739774 | BAL | Canada | 62740 | 62740 | 43153 |
| 8-SRR10739775 | BAL | Canada | 60512 | 60512 | 40677 |
| 8-SRR10739779 | BAL | Canada | 40539 | 40539 | 25706 |
| 8-SRR10739780 | NP | Canada | 76927 | 76927 | 46689 |
| 8-SRR10739781 | BAL | Canada | 72554 | 72554 | 46491 |
| 8-SRR10739783 | BAL | Canada | 58804 | 58804 | 36305 |
| 8-SRR10739785 | NP | Canada | 79545 | 79545 | 53342 |
| 8-SRR10739786 | BAL | Canada | 10351 | 10351 | 6772 |
| 8-SRR10739787 | NS | Canada | 80961 | 80961 | 47014 |
| 8-SRR10739791 | NS | Canada | 72308 | 72308 | 42294 |
| 8-SRR10739794 | OS | Canada | 96880 | 96880 | 58226 |
| 8-SRR10739795 | BAL | Canada | 54921 | 54921 | 35573 |
| 8-SRR10739797 | BAL | Canada | 13779 | 13779 | 9154 |
| 8-SRR10739798 | NS | Canada | 72310 | 72310 | 44197 |
| 8-SRR10739799 | BAL | Canada | 40738 | 40738 | 26290 |
| 8-SRR10739800 | NP | Canada | 72145 | 72145 | 43700 |
| 8-SRR10739801 | NS | Canada | 68195 | 68195 | 40842 |
| 8-SRR10739803 | BAL | Canada | 39573 | 39573 | 25454 |
| 8-SRR10739805 | NP | Canada | 99488 | 99488 | 68231 |
| 8-SRR10739807 | OS | Canada | 74177 | 74177 | 42036 |
| 8-SRR10739809 | NS | Canada | 83493 | 83493 | 53890 |
| 8-SRR10739812 | BAL | Canada | 51699 | 51699 | 32149 |
| 8-SRR10739813 | BAL | Canada | 53221 | 53221 | 32756 |
| 8-SRR10739815 | NS | Canada | 89690 | 89690 | 58038 |
| 8-SRR10739818 | OS | Canada | 87210 | 87210 | 59078 |
| 8-SRR10739819 | BAL | Canada | 76685 | 76685 | 48454 |
| 8-SRR10739821 | BAL | Canada | 76947 | 76947 | 51114 |
| 8-SRR10739824 | BAL | Canada | 20677 | 20677 | 12353 |
| 8-SRR10739825 | NP | Canada | 89191 | 89191 | 54147 |
| 8-SRR10739827 | NP | Canada | 65279 | 65279 | 41387 |
| 8-SRR10739828 | BAL | Canada | 7038 | 7038 | 4431 |
| 8-SRR10739829 | NS | Canada | 65877 | 65877 | 40688 |
| 8-SRR10739831 | BAL | Canada | 28607 | 28607 | 17840 |
| 8-SRR10739832 | BAL | Canada | 10106 | 10106 | 6327 |
| 8-SRR10739833 | NP | Canada | 81066 | 81066 | 50528 |
| 8-SRR10739834 | NP | Canada | 74716 | 74716 | 48619 |
| 8-SRR10739835 | NP | Canada | 52879 | 52879 | 33650 |
| 8-SRR10739838 | OS | Canada | 98329 | 98329 | 52136 |
| 8-SRR10739839 | NP | Canada | 79134 | 79134 | 50577 |
| 8-SRR10739840 | BAL | Canada | 35449 | 35449 | 22769 |
| 8-SRR10739841 | OS | Canada | 89296 | 89296 | 49462 |
| 8-SRR10739842 | BAL | Canada | 51669 | 51669 | 32532 |
| 8-SRR10739845 | BAL | Canada | 62576 | 62576 | 41906 |
| 8-SRR10739847 | BAL | Canada | 77038 | 77038 | 51247 |
| 8-SRR10739848 | NP | Canada | 75443 | 75443 | 46801 |
| 8-SRR10739849 | NS | Canada | 77396 | 77396 | 44043 |
| 8-SRR10739850 | NS | Canada | 76363 | 76363 | 49127 |
| 8-SRR10739851 | BAL | Canada | 35415 | 35415 | 22497 |
| 8-SRR10739852 | BAL | Canada | 58223 | 58223 | 38035 |
| 8-SRR10739853 | BAL | Canada | 35652 | 35652 | 22423 |
| 8-SRR10739856 | NS | Canada | 75740 | 75740 | 50262 |
| 8-SRR10739858 | BAL | Canada | 28504 | 28504 | 18094 |
| 8-SRR10739861 | OS | Canada | 75679 | 75679 | 45261 |
| 8-SRR10739862 | BAL | Canada | 20042 | 20042 | 12191 |
| 8-SRR10739867 | NP | Canada | 76581 | 76581 | 44618 |
| 8-SRR10739868 | OS | Canada | 80208 | 80208 | 44074 |
| 8-SRR10739869 | NS | Canada | 74548 | 74548 | 44557 |
| 8-SRR10739870 | BAL | Canada | 80199 | 80199 | 54093 |
| 8-SRR10739873 | OS | Canada | 77155 | 77155 | 46623 |
| 8-SRR10739877 | NS | Canada | 95517 | 95517 | 56341 |
| 8-SRR10739880 | BAL | Canada | 12492 | 12492 | 8000 |
| 8-SRR10739882 | BAL | Canada | 4345 | 4345 | 2829 |
| 8-SRR10739886 | BAL | Canada | 72851 | 72851 | 46775 |
| 8-SRR10739887 | BAL | Canada | 51575 | 51575 | 32311 |
| 8-SRR10739890 | BAL | Canada | 8059 | 8059 | 4957 |
| 8-SRR10739891 | NS | Canada | 86229 | 86229 | 56320 |
| 8-SRR10739893 | BAL | Canada | 57512 | 57512 | 37265 |
| 8-SRR10739894 | BAL | Canada | 65790 | 65790 | 41553 |
| 8-SRR10739895 | NS | Canada | 73660 | 73660 | 46838 |
| 8-SRR10739896 | NS | Canada | 91654 | 91654 | 58047 |
| 8-SRR10739898 | BAL | Canada | 81938 | 81938 | 47934 |
| 8-SRR10739899 | NP | Canada | 77907 | 77907 | 50196 |
| 8-SRR10739900 | BAL | Canada | 30229 | 30229 | 19789 |
| 8-SRR10739901 | BAL | Canada | 8913 | 8913 | 5655 |
| 8-SRR10739903 | NS | Canada | 85223 | 85223 | 54876 |
| 8-SRR10739904 | BAL | Canada | 65290 | 65290 | 41738 |
| 8-SRR10739907 | BAL | Canada | 78272 | 78272 | 50300 |
| 8-SRR10739908 | OS | Canada | 72783 | 72783 | 41803 |
| 8-SRR10739909 | NP | Canada | 79899 | 79899 | 52598 |
| 8-SRR10739911 | NP | Canada | 67784 | 67784 | 37828 |
| 8-SRR10739912 | OS | Canada | 52536 | 52536 | 29850 |
| 8-SRR10739913 | BAL | Canada | 60661 | 60661 | 38595 |
| 8-SRR10739914 | NP | Canada | 63899 | 63899 | 38971 |
| 8-SRR10739916 | OS | Canada | 85261 | 85261 | 47821 |
| 8-SRR10739917 | BAL | Canada | 45946 | 45946 | 28787 |
| 8-SRR10739919 | BAL | Canada | 60281 | 60281 | 37303 |
| 8-SRR10739920 | NP | Canada | 70421 | 70421 | 44396 |
| 8-SRR10739923 | NP | Canada | 70561 | 70561 | 43649 |
| 9-SRR15167368 | NS | USA | 204149 | 204149 | 180277 |
| 9-SRR15167370 | NS | USA | 87479 | 87479 | 74609 |
| 9-SRR15167371 | NS | USA | 84941 | 84941 | 55980 |
| 9-SRR15167376 | NS | USA | 4782 | 4782 | 3584 |
| 9-SRR15167377 | NS | USA | 148452 | 148452 | 102203 |
| 9-SRR15167378 | NS | USA | 53575 | 53575 | 41400 |
| 9-SRR15167379 | NS | USA | 2 | 2 | 2 |
| 9-SRR15167382 | NS | USA | 48607 | 48607 | 37580 |
| 9-SRR15167400 | NS | USA | 136738 | 136738 | 94718 |
| 9-SRR15167404 | NS | USA | 105162 | 105162 | 86818 |
| 9-SRR15167405 | NS | USA | 86923 | 86923 | 66238 |
| 9-SRR15167406 | NS | USA | 144597 | 144597 | 107754 |
| 9-SRR15167407 | NS | USA | 165538 | 165538 | 128561 |
| 9-SRR15167409 | NS | USA | 158422 | 158422 | 122212 |
| 9-SRR15167410 | NS | USA | 119789 | 119789 | 93844 |
| 9-SRR15167412 | NS | USA | 47751 | 47751 | 37575 |
| 9-SRR15167413 | NS | USA | 187517 | 187517 | 147787 |
| 9-SRR15167414 | NS | USA | 109657 | 109657 | 77458 |
| 9-SRR15167415 | NS | USA | 100240 | 100240 | 69461 |
| 9-SRR15167416 | NS | USA | 132391 | 132391 | 92663 |
| 9-SRR15167417 | NS | USA | 119288 | 119288 | 84218 |
| 9-SRR15167421 | NS | USA | 38740 | 38740 | 30916 |
| 9-SRR15167425 | NS | USA | 83617 | 83617 | 63603 |
| 9-SRR15167427 | NS | USA | 94308 | 94308 | 75057 |
| 9-SRR15167428 | NS | USA | 133811 | 133811 | 102449 |
| 9-SRR15167429 | NS | USA | 76999 | 76999 | 60079 |
| 9-SRR15167430 | NS | USA | 69702 | 69702 | 56464 |
| 9-SRR15167433 | NS | USA | 117831 | 117831 | 88573 |
| 9-SRR15167435 | NS | USA | 144810 | 144810 | 121159 |
| 9-SRR15167436 | NS | USA | 206022 | 206022 | 140263 |
| 9-SRR15167437 | NS | USA | 327739 | 327739 | 262403 |
| 9-SRR15167441 | NS | USA | 115754 | 115754 | 90906 |
| 9-SRR15167442 | NS | USA | 74301 | 74301 | 63862 |
| 9-SRR15167445 | NS | USA | 9743 | 9743 | 8538 |
| 9-SRR15167446 | NS | USA | 116644 | 116644 | 94158 |
| 9-SRR15167448 | NS | USA | 87044 | 87044 | 66584 |
| 9-SRR15167451 | NS | USA | 98927 | 98927 | 71833 |
| 9-SRR15167453 | NS | USA | 98531 | 98531 | 76791 |
| 9-SRR15167454 | NS | USA | 64030 | 64030 | 52034 |
| 9-SRR15167455 | NS | USA | 130762 | 130762 | 90962 |
| 9-SRR15167456 | NS | USA | 116204 | 116204 | 88528 |
| 9-SRR15167457 | NS | USA | 111784 | 111784 | 75913 |
| 9-SRR15167458 | NS | USA | 122342 | 122342 | 93765 |
| 9-SRR15167459 | NS | USA | 111931 | 111931 | 88518 |
| 9-SRR15167463 | NS | USA | 189943 | 189943 | 146920 |
| 9-SRR15167464 | NS | USA | 75813 | 75813 | 52585 |
| 9-SRR15167466 | NS | USA | 43156 | 43156 | 35061 |
| 9-SRR15167468 | NS | USA | 11844 | 11844 | 9510 |
| 9-SRR15167469 | NS | USA | 119186 | 119186 | 101150 |
| 9-SRR15167470 | NS | USA | 87756 | 87756 | 71554 |
| 9-SRR15167471 | NS | USA | 57299 | 57299 | 44916 |
| 9-SRR15167472 | NS | USA | 100979 | 100979 | 73491 |
| 9-SRR15167473 | NS | USA | 104734 | 104734 | 81896 |
| 9-SRR15167474 | NS | USA | 154709 | 154709 | 104071 |
| 9-SRR15167475 | NS | USA | 3280 | 3280 | 2435 |
| 9-SRR15167477 | NS | USA | 119167 | 119167 | 88823 |
| 9-SRR15167478 | NS | USA | 110805 | 110805 | 81059 |
| 9-SRR15167488 | NS | USA | 287873 | 287873 | 238920 |
| 9-SRR15167490 | NS | USA | 111501 | 111501 | 90201 |
| 9-SRR15167492 | NS | USA | 81654 | 81654 | 68242 |
| 9-SRR15167493 | NS | USA | 129937 | 129937 | 101737 |
| 9-SRR15167495 | NS | USA | 103614 | 103614 | 75780 |
| 9-SRR15167496 | NS | USA | 104324 | 104324 | 78360 |
| 9-SRR15167497 | NS | USA | 96640 | 96640 | 74225 |
| 9-SRR15167498 | NS | USA | 104547 | 104547 | 83419 |
| 9-SRR15167500 | NS | USA | 57763 | 57763 | 45418 |
| 9-SRR15167505 | NS | USA | 154063 | 154063 | 120937 |
| 9-SRR15167508 | NS | USA | 148102 | 148102 | 105716 |
| 9-SRR15167509 | NS | USA | 166838 | 166838 | 128680 |
| 9-SRR15167510 | NS | USA | 181007 | 181007 | 134273 |
| 9-SRR15167514 | NS | USA | 99406 | 99406 | 70557 |
| 9-SRR15167516 | NS | USA | 80656 | 80656 | 64760 |
| 9-SRR15167517 | NS | USA | 134510 | 134510 | 104672 |
| 9-SRR15167518 | NS | USA | 125161 | 125161 | 98463 |
| 9-SRR15167520 | NS | USA | 123559 | 123559 | 95178 |
| Ch-AL385-S05-01M0001 | NS | China | 79964 | 79925 | 53137 |
| Ch-AL385-S05-01M0002 | NS | China | 79780 | 79759 | 56875 |
| Ch-AL385-S05-01M0003 | NS | China | 79487 | 79453 | 57069 |
| Ch-AL385-S05-01M0004 | NS | China | 44226 | 44218 | 31239 |
| Ch-AL385-S05-01M0005 | NS | China | 63380 | 63365 | 42744 |
| Ch-AL385-S05-01M0006 | NP | China | 57579 | 57570 | 34085 |
| Ch-AL385-S05-01M0007 | NP | China | 73739 | 73724 | 51697 |
| Ch-AL385-S05-01M0008 | NP | China | 77514 | 77500 | 54864 |
| Ch-AL385-S05-01M0009 | NP | China | 49260 | 49251 | 31867 |
| Ch-AL385-S05-01M0010 | NP | China | 62477 | 62469 | 42240 |
| Ch-AL385-S05-01M0011 | OS | China | 79776 | 79764 | 55667 |
| Ch-AL385-S05-01M0012 | OS | China | 79633 | 79613 | 57555 |
| Ch-AL385-S05-01M0013 | OS | China | 80043 | 80014 | 54384 |
| Ch-AL385-S05-01M0014 | OS | China | 79963 | 79945 | 51953 |
| Ch-AL385-S05-01M0015 | OS | China | 79947 | 79922 | 53519 |
| Ch-AL385-S05-01M0016 | BAL | China | 79417 | 79404 | 51295 |
| Ch-AL385-S05-01M0017 | BAL | China | 79845 | 79833 | 51332 |
| Ch-AL385-S05-01M0018 | BAL | China | 78652 | 78639 | 55862 |
| Ch-AL385-S05-01M0019 | BAL | China | 79313 | 79296 | 60831 |
| Ch-AL385-S05-01M0020 | NS | China | 79818 | 79801 | 66009 |
| Ch-AL385-S05-01M0021 | NS | China | 79479 | 79467 | 57475 |
| Ch-AL385-S05-01M0022 | NS | China | 58169 | 58159 | 41403 |
| Ch-AL385-S05-01M0023 | NS | China | 68739 | 68719 | 51533 |
| Ch-AL385-S05-01M0024 | NS | China | 79567 | 79556 | 62306 |
| Ch-AL385-S05-01M0025 | NS | China | 72292 | 72286 | 59429 |
| Ch-AL385-S05-01M0026 | NS | China | 79171 | 79160 | 60607 |
| Ch-AL385-S05-01M0027 | NP | China | 72818 | 72802 | 58618 |
| Ch-AL385-S05-01M0028 | NP | China | 44384 | 44380 | 31060 |
| Ch-AL385-S05-01M0029 | NP | China | 74437 | 74422 | 53218 |
| Ch-AL385-S05-01M0030 | NP | China | 79921 | 79905 | 59759 |
| Ch-AL385-S05-01M0031 | NP | China | 79310 | 79297 | 58474 |
| Ch-AL385-S05-01M0032 | NP | China | 71046 | 71028 | 55786 |
| Ch-AL385-S05-01M0033 | NP | China | 78687 | 78680 | 62184 |
| Ch-AL385-S05-01M0034 | NP | China | 77236 | 77229 | 57952 |
| Ch-AL385-S05-01M0035 | OS | China | 79824 | 79811 | 50155 |
| Ch-AL385-S05-01M0036 | OS | China | 79842 | 79819 | 52901 |
| Ch-AL385-S05-01M0037 | OS | China | 72074 | 72057 | 53556 |
| Ch-AL385-S05-01M0038 | OS | China | 79618 | 79600 | 62257 |
| Ch-AL385-S05-01M0039 | OS | China | 80069 | 80048 | 63795 |
| Ch-AL385-S05-01M0040 | OS | China | 79858 | 79838 | 62329 |
| Ch-AL385-S05-01M0041 | OS | China | 79920 | 79901 | 53471 |

Supplemental Table S4 The significance of alpha diversity (Shannon Index) of the upper and lower respiratory tracts in different countries.

| Group 1 | Group 2 | Sample size | R | p-value |
| --- | --- | --- | --- | --- |
| Higher respiratory tract_Canada | Higher respiratory tract_China | 206 | 0.65 | 0.001 |
| Higher respiratory tract_Canada | Higher respiratory tract_Italy | 179 | -0.04 | 0.647 |
| Higher respiratory tract_Canada | Higher respiratory tract_USA | 251 | 0.22 | 0.001 |
| Higher respiratory tract_Canada | Lower respiratory tract_Canada | 274 | 0.28 | 0.001 |
| Higher respiratory tract_Canada | Lower respiratory tract_China | 173 | 0.66 | 0.001 |
| Higher respiratory tract_Canada | Lower respiratory tract_Italy | 187 | 0.29 | 0.001 |
| Higher respiratory tract_Canada | Lower respiratory tract_USA | 172 | 0.62 | 0.001 |
| Higher respiratory tract_China | Higher respiratory tract_Italy | 47 | 0.63 | 0.001 |
| Higher respiratory tract_China | Higher respiratory tract_USA | 119 | 0.77 | 0.001 |
| Higher respiratory tract_China | Lower respiratory tract_Canada | 142 | 0.75 | 0.001 |
| Higher respiratory tract_China | Lower respiratory tract_China | 41 | 0.30 | 0.011 |
| Higher respiratory tract_China | Lower respiratory tract_Italy | 55 | 0.95 | 0.001 |
| Higher respiratory tract_China | Lower respiratory tract_USA | 40 | 0.66 | 0.002 |
| Higher respiratory tract_Italy | Higher respiratory tract_USA | 92 | 0.62 | 0.001 |
| Higher respiratory tract_Italy | Lower respiratory tract_Canada | 115 | 0.14 | 0.081 |
| Higher respiratory tract_Italy | Lower respiratory tract_China | 14 | 0.84 | 0.003 |
| Higher respiratory tract_Italy | Lower respiratory tract_Italy | 28 | 0.56 | 0.001 |
| Higher respiratory tract_Italy | Lower respiratory tract_USA | 13 | 0.81 | 0.004 |
| Higher respiratory tract_USA | Lower respiratory tract_Canada | 187 | 0.61 | 0.001 |
| Higher respiratory tract_USA | Lower respiratory tract_China | 86 | 0.92 | 0.001 |
| Higher respiratory tract_USA | Lower respiratory tract_Italy | 100 | 0.80 | 0.001 |
| Higher respiratory tract_USA | Lower respiratory tract_USA | 85 | 0.82 | 0.002 |
| Lower respiratory tract_Canada | Lower respiratory tract_China | 109 | 0.59 | 0.001 |
| Lower respiratory tract_Canada | Lower respiratory tract_Italy | 123 | -0.06 | 0.788 |
| Lower respiratory tract_Canada | Lower respiratory tract_USA | 108 | 0.68 | 0.001 |
| Lower respiratory tract_China | Lower respiratory tract_Italy | 22 | 0.94 | 0.001 |
| Lower respiratory tract_China | Lower respiratory tract_USA | 7 | 0.67 | 0.029 |
| Lower respiratory tract_Italy | Lower respiratory tract_USA | 21 | 0.96 | 0.002 |

Supplemental Table S5 The statistics of the beta diversity (Bray-Curtis distance) of different niches in different countries.

| Pairwise ANOSIM results for Canada | | | | |
| --- | --- | --- | --- | --- |
| Group 1 | Group 2 | Sample size | R | p-value |
| BAL | NP | 50 | 0.11 | 0.008 |
| BAL | NS | 50 | 0.36 | 0.001 |
| BAL | OS | 50 | 0.57 | 0.001 |
| NP | NS | 56 | 0.05 | 0.042 |
| NP | OS | 56 | 0.54 | 0.001 |
| NS | OS | 56 | 0.48 | 0.001 |
| Pairwise ANOSIM results for China | | | | |
| Group 1 | Group 2 | Sample size | R | p-value |
| BAL | NP | 14 | 0.39 | 0.006 |
| BAL | NS | 14 | 0.29 | 0.053 |
| BAL | OS | 14 | 0.71 | 0.003 |
| NP | NS | 20 | -0.07 | 0.915 |
| NP | OS | 20 | 0.25 | 0.017 |
| NS | OS | 20 | 0.22 | 0.018 |
| Pairwise anosim results for Italy | | | |  |
| Group 1 | Group 2 | Sample size | R | p-value |
| BAL | NS | 18 | 0.53 | 0.001 |
